# Improving lipid identification and quantification: chromatogram deconvolution for LC-MS/MS workflows

**DOI:** 10.1093/bioinformatics/btag695

**Published:** 2026-09-18

**Authors:** Felix Niedermaier, Denise Wolrab-Frühauf, Robert Ahrends, Dominik Kopczynski

**Affiliations:** Department of Analytical Chemistry, Faculty of Chemistry, University of Vienna, Währinger Straße 38–42, 1090 Vienna, Vienna, Austria; Vienna Doctoral School in Chemistry (DoSChem), University of Vienna, Währinger Straße 42, 1090 Vienna, Vienna, Austria; Department of Analytical Chemistry, Faculty of Chemistry, University of Vienna, Währinger Straße 38–42, 1090 Vienna, Vienna, Austria; Department of Analytical Chemistry, Faculty of Chemistry, University of Vienna, Währinger Straße 38–42, 1090 Vienna, Vienna, Austria; Department of Analytical Chemistry, Faculty of Chemistry, University of Vienna, Währinger Straße 38–42, 1090 Vienna, Vienna, Austria

## Abstract

**Motivation:**

Lipidomics relies on mass spectrometry-based workflows to identify and quantify complex lipid species. Due to the modular architecture of lipids, including headgroups, backbones, and fatty acyl chains, distinct precursor ions often produce isobaric or identical fragment ions. This problem is amplified in data-independent acquisition (DIA), where wide isolation windows (e.g. 25 Da) allow co-eluting precursors with different *m/z* values to generate highly chimeric MS/MS spectra. Consequently, fragments originating from multiple precursors, including isobars, isomers, and lipid-class-specific ions, are merged into a single MS/MS spectrum. Current lipid identification strategies often process such chimeric spectra in an uncontrolled manner, assigning them to one or more candidate lipids, thereby increasing false-positive identifications.

**Results:**

Here, we introduce an algorithm that deconvolutes chimeric MS/MS spectra and chromatograms by exploiting their temporal correlation with associated precursor chromatographic profiles, independent of elution peak shape. Using simulated and real experimental lipidomics data, we demonstrate that this approach substantially improves lipid fragment assignment, reduces false-positive identifications, and enables more reliable fragment-level quantification, leading to more robust downstream statistical analyses and biological interpretations.

**Availability and Implementation:**

Source code of the software library: GitLab (Apache 2.0 License): https://gitlab.com/computational-multiomics/projects/mixture-model-deconvolution; Data: Zenodo (Apache 2.0 License): https://doi.org/10.5281/zenodo.21218594.

## 1 Introduction

Lipids constitute a large chemical space within the metabolome and play essential roles in membranes, signaling, and energy provision in cells (van Meer *et al.* 2008, Peng *et al.* 2018, de Jonckheere *et al.* 2023). Unlike linear biopolymers, such as DNA, RNA, and proteins, lipids are branched and are assembled from modular building blocks such as fatty acyl chains, backbone structures, and headgroups (Peng and Ahrends 2016). In tandem mass spectrometry (MS/MS), precursor ions are isolated by their mass-to-charge ratio and dissociated, typically by collision-induced dissociation (CID), yielding fragments whose masses reflect the building blocks and their connectivity. The chemical connectivity between these building blocks, such as ester, ether, vinyl ether, amide, or phosphodiester bonds, defines the physicochemical fragmentation behavior, causing all lipids within a class to follow the class’s characteristic fragmentation pattern, with fragment abundances depending on the collision energy (Peng *et al.* 2020). If the fragmentation behavior of a lipid is known, it can be identified in MS/MS experiments using rule-based models searching for its specific fragments to determine the class and species (Herzog *et al.* 2011, 2012, Peng and Ahrends 2016, Peng *et al.* 2020).

Liquid chromatography coupled to high-resolution tandem mass spectrometry (LC–MS/MS) is a primary analytical workflow for lipid analysis. Today, most studies applying a separation method rely on data-dependent acquisition (DDA), in which precursor ions are stochastically selected for fragmentation in top-*n* experiments based on signal intensity. While DDA produces high-quality MS^2^ spectra when triggered at the apex of the precursors’ elution profile, it inherently biases the acquisition toward highly abundant ions, leading to limited reproducibility due to incomplete coverage and unstable precursor selection across runs. In contrast, data-independent acquisition (DIA), which is well established in proteomics, systematically fragments all ions within predefined isolation windows, providing an untargeted, unbiased, and highly reproducible alternative with the potential to substantially improve lipid detection and quantification across many samples. Often, concepts from proteomics, such as a target-decoy model for applying a false discovery rate, retention time prediction, or systematic fragment computation, would offer significant added value but cannot be transferred directly to lipidomics without accounting for the chemical nature of these non-linear branched structures (Peng and Ahrends 2016, Peng *et al.* 2020, Kretschmer *et al.* 2024).

Despite these benefits, DIA-LC-MS/MS has not yet been widely adopted in lipidomics (Köfeler *et al.* 2021, Li and Lämmerhofer 2021). This is mainly due to the greater structural complexity of lipids compared with the linear, continuous alignment of amino acids in proteins. The co-fragmentation of isomeric and isobaric lipid species (Kopczynski *et al.* 2024) into a single fragment spectrum further complicates the interpretation of already chimeric MS^2^ data. The reliance on class-specific fragmentation patterns poses an issue for structural resolution, since co-eluting lipids of the same class frequently share fragments and, in some cases, even different classes do so, as in fatty acid losses. In DIA, fragments from multiple co-isolated precursors are recorded within the same spectrum, increasing the risk of misidentifications, since fragments may be assigned to the wrong precursor. Further, lipidomics often relies on fragment chromatograms for quantification, meaning that fragments shared between co-eluting species can distort the quantitative accuracy as well. DIA MS^2^ chromatogram deconvolution is not a priority in proteomics workflows, since the probability that two precursors in an LC proteomics experiment create a convoluted MS^2^ chromatogram is low, as they would have to share a fragment, co-elute and co-fragment within the same isolation frame. Existing DIA analysis tools, such as DIA-Umpire (Tsou *et al.* 2015), therefore assign MS^2^ chromatograms to a single precursor chromatogram based on statistical correlation. This challenge would be alleviated by dedicated lipidomics software that can extract and deconvolute such spectra and assign fragment ion chromatograms to their corresponding precursors in a statistically robust manner. Existing DIA analysis tools, largely optimized for proteomics, either rely on predefined target lists or make strong assumptions about chromatographic peak shapes and elution behavior. For example, DecoMetDIA defines model peak shapes (Yin *et al.* 2019), the GoldenHaystack (GH) algorithm, exploiting that pairs of peptides coeluting exactly in one subset of LC-MS files do not necessarily coelute exactly in another subset, searches multiple replicates for measurements in which the two precursors are separable and transfers this ratio to the convoluted measurements (Saxena *et al.* 2026), and CorrDec correlates signals across repeated measurements (Tada *et al.* 2020). Such assumptions may not hold in lipidomics due to heterogeneous chromatographic behavior, chimeric spectra, and complex co-elution patterns.

Here, we present a novel algorithm that enables de-novo DIA analysis for lipidomics by deconvoluting MS^2^ chromatograms and assigning fragment ions to precursor candidates solely based on statistical relationships. The method operates without target lists, prior knowledge of lipid identities, correlations over multiple measurements, or assumptions about chromatographic peak shapes or elution profiles. Instead, it relies on a single, minimal assumption: that the intensities and elution profiles of lipid precursors and their corresponding fragment ions are correlated. By exploiting this principle, the algorithm disentangles complex DIA spectra and reconstructs precursor-fragment relationships directly from the data.

The algorithm is implemented as a modular software library designed for easy integration into existing lipidomics pipelines, tools, and workflows. By lowering the methodological and computational barriers to DIA-based lipidomics, this work aims to facilitate broader adoption of unbiased acquisition strategies and unlock the full potential of DIA-LC-MS/MS for comprehensive lipidome analysis.

## 2 Materials and methods

A single fragment ion can originate from multiple precursors, when the precursors’ chromatographic peaks are not fully resolved, the fragment forms a composite shape along the retention time axis. To correctly quantify this fragment for each precursor, the composite chromatogram must be separated into individual contributions that follow the elution profile of each precursor before integration. A direct comparison of absolute signal intensities between precursors and fragments is not possible because different molecules ionize with different efficiencies and produce fragments with different intensities. Therefore, we abstract the problem by modelling the fragment chromatogram as a weighted mixture model of multiple source distributions, corresponding to the precursor chromatograms. By fitting this mixture model, we obtain, for each measured point in the fragment chromatogram, posterior likelihoods that indicate the probabilistic contribution of each precursor to the measured point.

The approach imposes requirements on the measurement method. It requires one dimension in which the precursor and its fragments correlate; for DIA-LC-MS/MS, this is the retention time. Since fragments are produced after elution, their profiles mirror the precursor’s elution profile. For our proposed method, all isolation windows and their *m/z* boundaries must be precisely the same in consecutive DIA cycles. As such, DIA-LC-MS/MS produces continuous chromatograms in MS^2^. Figure 1 shows an example workflow, i.e. compatible with this deconvolution method. Stable MS^2^ isolation windows of 25 Da with DIA were specified here for this example and later for the dataset acquisition and method validation, but can vary depending on the acquisition speed.

**Figure 1:**
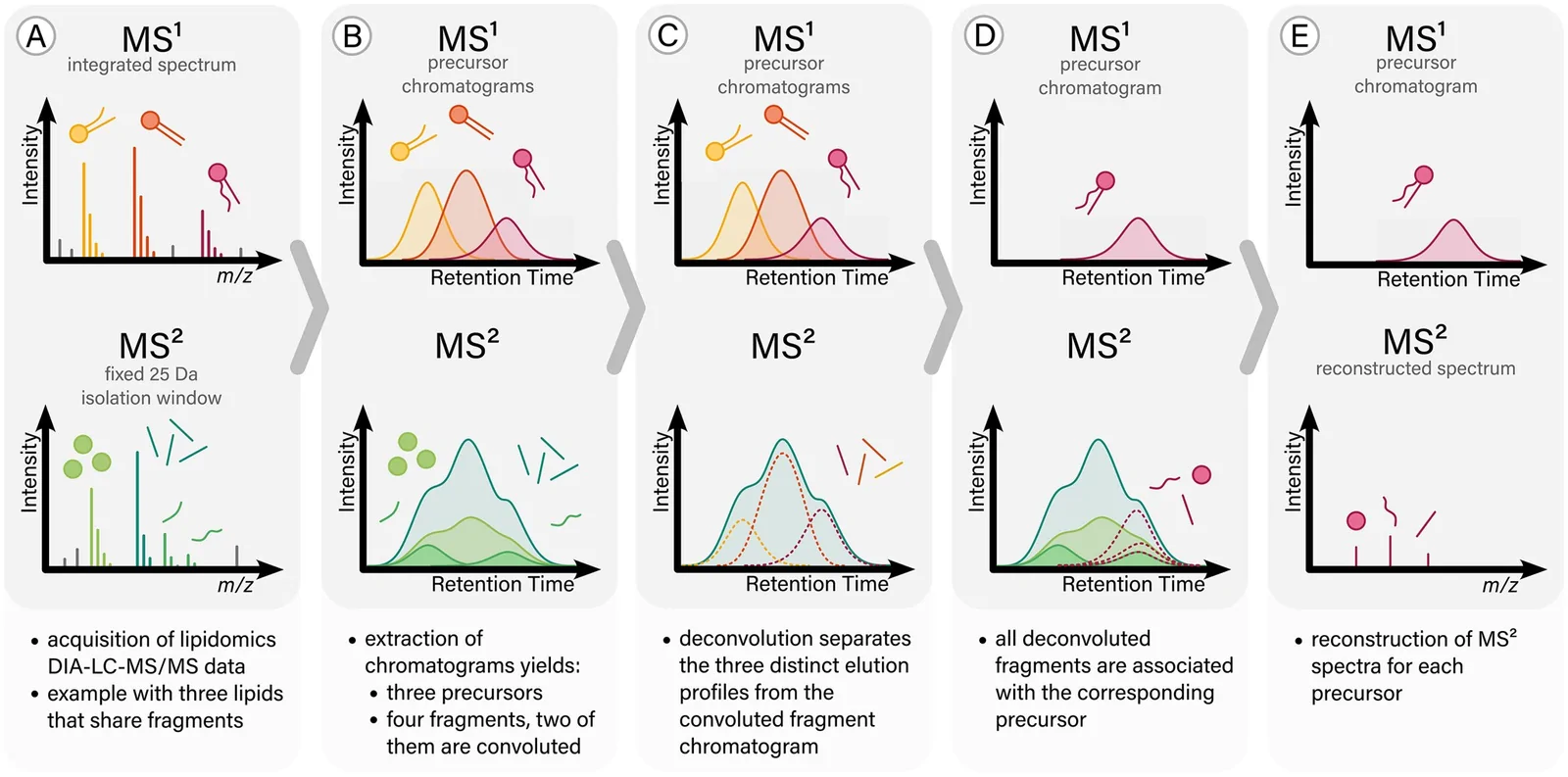
Chromatogram deconvolution workflow from raw lipidomics DIA-LC-MS/MS data towards reconstructed fragments. (A) Measured data, a fixed-size and position frame isolation window is required to form fragment chromatograms in MS^2^. In this example, three precursors are co-isolated within the same isolation window, sharing two fragments. Stable MS^2^ isolation windows of 25 Da with DIA were specified here for this example, but can vary depending on the acquisition speed. (B) Chromatograms are extracted from the spectra over time. These shared fragments form convoluted elution peaks. (C) Deconvolution of one shared fragment using the precursor elution profiles. The fragment elution profile is split into three parts, one for each precursor. (D) Deconvolution is executed for all fragments and precursors. Therefore, for each precursor, all its fragments are reassigned. (E) Reconstruction of the clean MS^2^ spectrum for one exemplary precursor without fragments from co-eluting precursors.

The workflow starts with an LC-MS/MS measurement, with all spectra converted to centroid mode; a single *m/z* and intensity data point is defined as a signal. First, signals are connected into continuous chromatograms, separately for MS^1^ and each MS^2^ isolation window. We implemented this using a variant of the Needleman-Wunsch algorithm (Needleman and Wunsch 1970) that aligns peaks by mass differences rather than matches and mismatches. We chose this over simple *m/z* binning because Needleman-Wunsch enforces a one-to-one assignment between signals in consecutive spectra and allows gaps of adjustable size. In the second step, for each MS^2^ isolation window and all its associated MS^2^ fragment chromatogram, candidate MS^1^ precursor chromatograms are assigned exclusively according to their retention time and isolation window boundaries. The chromatograms are indexed in an interval tree (de Berg *et al.* 2008) to perform this matching efficiently, since the search for overlapping MS^1^ precursor chromatograms is repeated for each MS^2^ fragment chromatogram. We call a single MS^2^ curve and a set of candidate MS^1^ curves a convoluted system. Note that a precursor chromatogram may appear in several convoluted systems.

The consecutive deconvolution step requires that both the MS^2^ chromatogram and all its candidate MS^1^ chromatograms must be aligned to the same retention time points within the convoluted system. Since MS^1^ and the associated MS^2^ spectra are not recorded exactly simultaneously, precursor chromatograms are linearly interpolated and cropped to the retention time values of the measured fragment chromatogram curve. After interpolation, retention time values no longer need to be considered. Since several MS^2^ curves may have the same candidate MS^1^ curve, for each MS^2^ curve, the MS^1^ retention time alignment is done individually. Each convoluted system can then be deconvoluted independently.

### 2.1 Application of the deconvolution algorithm

The core premise of the algorithm is, as stated, that the elution profile of an analyte (MS^1^ chromatogram) is identical to its fragments’ elution profile (MS^2^ chromatograms). Rather than presuming a particular curve shape, the algorithm considers each candidate MS^1^ chromatogram as a model in a convoluted system, representing the curve intensities as the corresponding probability distributions. Let *k* be the number of models (MS^1^ chromatograms) in a convoluted system, where i∈{1,…,k} and *n* is the number of data points for all curves, where j∈{1,…,n}. In a first step, the curve intensities need to be normalized for each model *i* to obtain probability distributions $p_{i}$ (Equation 1) that follow:


(1) $$\sum_{j=1}^{n}p_{i,j}=1\;\text{for}\;\text{each}\;i\in \{1,\ldots ,k\}.$$

The algorithm interprets the measured fragment chromatogram $p_{F}$ (Equation 2) in the convoluted system as the result of a mixture model:


(2) $$p_{F,j}=w_{0}+\sum_{i=1}^{k}w_{j}p_{i,j}$$

for each j∈{1,…,n} where $0\leq w_{i}\leq 1$ is the weight of model *i* and $w_{0}$ is a background weight for all the signals in the fragment curve that cannot be explained by any precursor curve. The following applies: $\sum_{i=0}^{k}w_{i}=1$.

To find the optimal parameters $w_{i}$, we applied the Expectation Maximization (EM) algorithm (Dempster *et al.* 1977), which is commonly used to estimate parameters of stochastic models (Sammut and Webb 2010, Kopczynski and Rahmann 2015). The algorithm iteratively alternates between two steps (Equations 3 and 5) to optimize the weights until a convergence criterion is met. In the first (expectation) step, values $\gamma_{i,j}^{\left(t\right)}$ are computed to quantify the likelihood of a fragment curve data point *j* being explained by model *i* in iteration *t*. Let


(3) $$\gamma_{i,j}^{\left(t\right)}=\frac{w_{i}^{\left(t\right)}p_{i,j}}{\sum_{m=0}^{k}w_{m}^{\left(t\right)}p_{m,j}}$$

for each i∈{0,…,k} and j∈{1,…,n}. Good initialization of the weights (Equation 4) is important to keep the number of iterations as low as possible and to reduce the probability of the algorithm converging towards a local maximum instead of the global maximum:


(4) $$w_{i}^{\left(0\right)}\propto \int p_{i},\;\text{with}\;i\in \{1,\ldots ,k\}\;\text{and}\;w_{0}^{\left(0\right)}=\epsilon$$

In the second (maximization) step (Equation 5), the weights are updated based on the previously estimated likelihoods, let


(5) $$w_{i}^{\left(t+1\right)}=\sum_{j=1}^{n}\gamma_{i,j}^{\left(t\right)},\;\text{with}\;\sum_{i=0}^{k}w_{i}^{\left(t+1\right)}=1.$$

Convergence is assumed when the weights show no significant change between successive iterations *t* and t+1. The final likelihoods are now used to divide the fragment curve into k+1 curves: the *k* curves correspond to the shape of the precursor curves and reflect the clean (i.e. deconvoluted) fragment chromatograms provided by the precursors. The resulting $w_{0}$ model represents all signal components that could not be assigned and and may therefore be excluded from further analysis.

### 2.2 Evaluation on simulated convoluted systems

To evaluate the algorithm, we distinguish between two scenarios: in the first one, we compare simulated data before convolution and after reconstruction by using a score function $s_{c}$ (combined score, Equation 8); and in the second scenario, for measured lipidomics data, we compare the similarity of MS^1^ chromatograms with their reassigned deconvoluted MS^2^ chromatograms by applying a score function $s_{s}$ (shape score, Equation 6).

For the evaluation on simulated convoluted systems we randomly generate a set of overlapping Gaussian distribution curves that resemble a DIA-LC-MS/MS measurement to showcase how the algorithm deconvolutes such a system and where the mathematical limits of the approach are.

To simulate a convoluted system, first, two retention time vectors are generated from measurement frequencies for both MS^1^ and MS^2^. Then, *k* normal distributions with random mean, standard deviation, and scalar are generated, and their probability distribution function is sampled at the MS^1^ retention time vector; these represent the precursor chromatograms. The *k* precursor chromatograms are interpolated at the MS^2^ retention time vector and multiplied by a random fragmentation efficiency factor between 0 and 1, representing the theoretical fragment chromatograms. Random noise is added to both the precursor chromatograms and theoretical fragment chromatograms independently. The *k* noisy theoretical fragment chromatograms are summed up at each retention time to calculate the noisy convoluted measured fragment chromatogram. Finally, the deconvolution is executed on the precursor chromatograms and the measured fragment chromatogram, constituting a convoluted system.

When assessing the deconvolution of simulated convoluted systems, we are exploiting the fact that we initially construct theoretical MS^2^ chromatograms for each MS^1^ chromatogram in a deconvolution system and consecutively overlap all MS^2^ chromatograms into one convoluted MS^2^ chromatogram. Thus, we are able to quantitatively compare deconvoluted MS^2^ chromatograms with the original ones. For this purpose, we use (i) the cosine similarity (Equation 6), which quantifies the similarity of two -dimensional vectors, and (ii) the ratio between two areas under their chromatograms (Equation 7). In our case, we consider the chromatogram curves as vectors in an *n*-dimensional space along the retention time axis, with each dimension corresponding to an intensity value. Therefore, to calculate the cosine similarity, it is again necessary to align a chromatogram pair to the same retention time vector. For a fair comparison, the retention times are sampled uniformly for interpolation from the minimum of both chromatograms to the respective maximum of both (see also Fig. 2A).


(6) $$s_{s}(\vec{a},\vec{b})=\frac{\sum_{j=1}^{n}a_{j}b_{j}}{\sqrt{\sum_{j=1}^{n}a_{i}^{2}}\sqrt{\sum_{j=1}^{n}b_{j}^{2}}}$$

**Figure 2:**
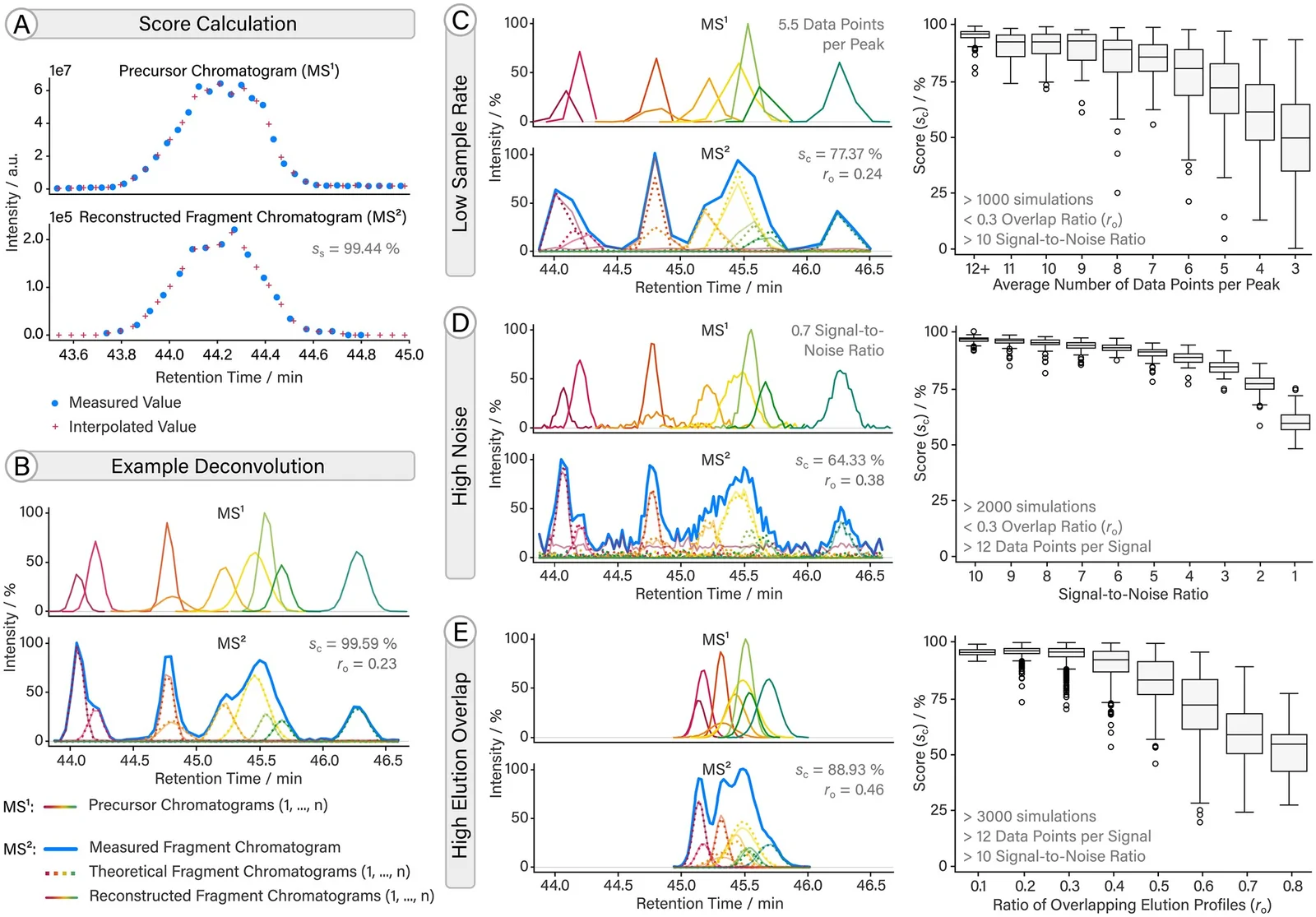
Effect of variable conditions on algorithm performance. (A) Illustration of the shape score calculation on a measured precursor and fragment chromatogram pair (their measured signals are shown as blue marks). The cosine similarity requires two vectors of equal size, thus interpolation is performed to a shared retention time vector (red marks). For a fair comparison, the retention times for interpolation are sampled uniformly from the minimum of both chromatograms to their maximum. (B) Deconvolution performed on an example set of simulated precursors that, together with varying fragmentation efficiency, produce one shared fragment chromatogram. Upper: simulated precursor chromatograms; lower: theoretical fragment chromatograms (dashed lines with associated colors) with varying fragmentation efficiencies. Overlapping them simulates a measured convoluted fragment chromatogram (blue line). The predicted fragment contributions can be compared directly with the theoretical fragment contributions, since we know the ground-truth fragmentation efficiency for all precursors in a simulated example. The same simulated convoluted system is deconvoluted again, whereby one of three different conditions has been modulated: (C) Algorithm performance with varying sample rate of the chromatograms. Fewer data points per chromatogram correlate with lower prediction performance, since interpolation loses accuracy. Robust performance is observed when the average number of data points per peak exceeds 5. (D) Robustness to noise, in an MS/MS experiment, the noise between MS1 and MS2 is asymmetric; a signal-to-noise ratio of higher than 1 should be ensured. (E) Effect of precursor chromatogram overlap over the retention time on the prediction performance; complete co-elution of precursors with identical peak shapes represents a non-identifiable case for the algorithm, but with slight separation or peak width difference, the algorithm is able to separate them.

Under cosine similarity, the optimum corresponds to both curves having identical shapes, whereas the worst case occurs when one chromatogram ends before the other begins. The cosine similarity ranges from −1 to 1, but since the chromatogram intensity values are only positive, the similarity score is within 0 to 1, whereby 0 means total inequality and 1 means total equality. Since cosine similarity measures the angle between two vectors, the similarity value is independent of the chromatogram’s scaling. The scaling is important, however, since by using solely $s_{s}$ (shape score, Equation 6), we would account for the relative chromatogram shape, but not for their absolute areas. Therefore, we additionally use the ratio of the absolute integrals of two chromatograms between 0 and 1 as score $s_{w}$ (weight score, Equation 7). Interpolation is not required since we use the chained trapezoidal rule to calculate the integrals (Kalambet *et al.* 2018).


(7) $$s_{w}(\vec{a},\vec{b})=\frac{\text{min}(\int \vec{a},\int \vec{b})}{\text{max}(\int \vec{a},\int \vec{b})}$$

The two similarity scores $s_{s}$ and $s_{w}$ (Equations 6 and 7) are then multiplied to express a combined similarity score $s_{c}$ (combined score, Equation 8) evaluating the shape and the weight of a chromatogram pair. When evaluating a whole convoluted system, we calculate the weighted arithmetic mean over all *k* pairs of theoretical and deconvoluted fragment chromatograms $c_{T}=(\vec{c_{T,1}},\ldots ,\vec{c_{T,k}})$ and $c_{D}=(\vec{c_{D,1}},\ldots ,\vec{c_{D,k}})$, weighted by the integrals of the theoretical fragment chromatograms:


(8) $$s_{c}(c_{T},c_{D})=\frac{\sum_{i=1}^{k}s_{s}(\vec{c_{T,i}},\vec{c_{D,i}})s_{w}(\vec{c_{T,i}},\vec{c_{D,i}})\int \vec{c_{T,i}}}{\sum_{i=1}^{k}\int \vec{c_{T,i}}}.$$

To quantify how strongly the precursor elution profiles overlap in a convoluted system, we define an overlap ratio (Supplementary Data Section 1, Equation S3; see also Fig. 2E): 0 % if two curves do not overlap, 100 % if two curves overlap exactly, and 200 % if three curves overlap exactly.

Further insights on the methodological choices are provided in the Supplementary Data Section 1.

### 2.3 Evaluation on measured data

Since we do not know the underlying distributions of the fragment contributions in measured data, we cannot apply the weight score $s_{w}$ (Equation 7), but we can compare the shape of the deconvoluted chromatogram with the precursor chromatogram using the cosine similarity $s_{s}$ (Equation 6) to qualitatively evaluate the deconvolution results. Further, for a convoluted system with identified precursors and a comprehensive set of known associated fragments, we can compare the known associated fragment chromatograms with all MS^2^ chromatograms during the elution times of the precursors. If deconvolution is successful, the intensities of non-associated MS^2^ chromatograms should be zero, the intensity of shared fragments should be reduced, and the intensity of unique fragments should remain the same as before deconvolution for each precursor.

To illustrate the behavior of the proposed deconvolution workflow on real data, we analysed a representative LC-MS/MS measurement of Rat (*Rattus norvegicus*) brain synaptic junction samples using reversed-phase DIA-MS on a Vanquish Horizon UHPLC system (Thermo Scientific, Bremen, Germany) coupled to a Q Exactive HF mass spectrometer (Thermo Scientific, Bremen, Germany). The synaptic junction extraction process is described by Wendholt *et al.* (2006). Protein precipitation was used for sample preparation. Therefore, 250 µL of ice cold MeOH was added to the rat brain synaptic junction samples isolated from rats (CxSJ180723, 300 µg, 4 aliquotes). The rat brain synaptic junction samples were homogenized by ultrasonicating the samples for 5 min followed by freezing with liquid nitrogen for 30 seconds. This step was repeated for three times before adding the internal standard mixture and 250 µL BuOH. The samples were vortexed and sonicated for 5 min. After 30 min of shaking at 4°C on the thermomix, the samples were centrifuged for 10 min at 12000×g, The supernatant was transferred in new Eppendorf tubes and evaporated under nitrogen. The residue was dissolved in 50 µL CHCl_3_/MeOH 1/1, for 20 seconds, vortexed, ultrasonicated, centrifuged for 5 min and the supernatant transferred into HPLC vials for measurements with a Vanquish Horizon UHPLC system (Thermo Scientific, Bremen, Germany) coupled to a Q Exactive HF mass spectrometer (Thermo Scientific, Bremen, Germany). A 90 min run using a reversed phase column (ACQUITY UPLC BEH C18 Column, 130 Å, 1.7 µm, 2.1 mm × 150 mm) was employed. A flow rate of 0.25 mL/min was set with a total injection volume of 1 or 5 µL, respectively. The injection needle was washed using a mixture of MeOH/ACN/IPA/H_2_O (1:1:1:1, v/v/v/v) after each injection. The mobile phase A consisted of ACN/H_2_O (30/70) and 0.1 % FA, while the mobile phase B was 9/1/90 ACN/H_2_O/IPA with 10 mM NH_4_FA and 0.1 % FA. The gradient was as follows: 0 min 50 % B, 70 min 99 % B, kept at 99 % B for 15 min for washing and at 85 min back flushed to starting conditions 50 % B until 90 min. For the full MS^1^ scan, the resolution was set to 30 000 the AGC target to $1\times 10^{6}$, and the scan range to *m/z* 200–1500. For the MS/MS scans, the isolation window was *m/z* 25.0, the resolution to 15 000 the AGC target was $2\times 10^{5}$, the normalized collision energy (NCE) was set to 30, and centroid spectra were acquired. To ensure a sufficient number of data points across each chromatographic peak, the DIA isolation windows were adjusted according to retention time. Specifically, isolation windows covering precursor ions below *m/z* 500 were acquired only during the first 45 min of the run, whereas isolation windows covering precursor ions above *m/z* 1000 were acquired only after 45 min. The measurement was manually annotated to qualitatively evaluate the fragment deconvolution approach.

### 2.4 Comparison with existing tools

To complement the real measurement, we simulated DIA lipidomics datasets using the lipids identified in the measured dataset, with fragmentation patterns generated from LipidCreator (Peng *et al.* 2020) and normal plus lognormal peak shapes with tailing to simulate realistic chromatographic behavior. Simulating the data allowed us to obtain realistic fragment overlaps from a real measurement while retaining an exact ground-truth for evaluation. Three experiments were performed to assess identification and quantification performance. For these experiments, precursors are classified into four overlap scenarios. OS1: precursors with no overlap; OS2: precursors with MS^2^ overlap, meaning they co-elute with at least one other precursor within the same DIA isolation window and share at least one fragment with it; OS3: precursors with MS^1^ overlap, meaning they co-elute with at least one other precursor of the same *m/z* within a given ppm accuracy, without sharing any fragments; and OS4: precursors with MS^1^ & MS^2^ overlap, meaning they co-elute with at least one other precursor of the same *m/z* within a given ppm accuracy and additionally share at least one fragment with it.

We compared our method against MS-DIAL 5 (Takeda *et al.* 2024), version v5.5.260323, the current state-of-the-art DIA mass spectrometry analysis tool, which implements model-based peak-shape deconvolution for single experiments and correlation-based deconvolution across measurement files. Both methods take an mzML file as input and output an MGF file containing the reconstructed MS^2^ spectra for each precursor.

To compare identification performance, we simulated 10 runs with a 50 Da isolation window and 0.5 % jitter in the analyte abundance, and evaluated the cosine similarity between all simulated ground-truth MS^2^ spectra and their respective reconstructed MS^2^ spectra for each precursor across the 10 runs. Statistical significance of the difference between both methods in cosine similarity distribution mean was determined with a paired nonparametric permutation test using Monte Carlo resampling (Fisher 1935, Metropolis *et al.* 1953, Virtanen *et al.* 2020). To compare quantification performance, we simulated 10 runs with random abundances for each analyte, uniformly distributed between 0.1× and 10× of the analyte abundance, again using a 50 Da isolation window, and compared the abundance of each fragment in the ground-truth against its reconstructed abundance. The quality of this response was determined using the R^2^ value between ground-truth and reconstructed abundance. Finally, to assess quantification fold-change stability, we simulated 10 runs for each combination of abundance factors [1×, 1.5×, 2×, 5×, 10×] and isolation window sizes [5, 25, 50, 100, 1300] Da, where the 1300 Da window corresponds to AIF (All Ion Fragmentation), with 0.5 % jitter of the analyte abundance. Here, a realistic quantification approach was used, where for each precursor only fragments from the ground-truth that occurred in every measurement were selected and summed into a single abundance value. Statistical significance between the results of the two methods was determined for the difference in mean and variance of the analyte abundance log_2_ fold change distributions (see also Fig. 4C1), and for the difference in mean and variance of the log_2_ fold change error distributions (see also Fig. 4C2), each via a paired nonparametric permutation test using Monte Carlo resampling.

### 2.5 Runtime benchmark

The algorithm has a computational complexity of when applied to a convoluted system, where is the number of precursors and the number of data points for all curves as above.

We did a runtime benchmark based on the number of precursors per deconvolution and ran an empirical complexity fit for the exponent via nonlinear least squares and tried to isolate a potential log growth component, which confirmed a 1.01±0.001 exponent in line with the linear growth and no log growth component between 1 and 2048 precursors. Further details on the benchmark are provided in the Supplementary Data Section 2.

## 3 Results

The results presented here demonstrate the performance of the deconvolution workflow on simulated data with known fragment contributions as well as on a manually annotated DIA-LC-MS/MS measurement. Two conceptually distinct simulation approaches were used: a convoluted system simulation of overlapping Gaussian elution profiles to probe the algorithm’s behavior under controlled, systematically varied conditions (Fig. 2), and realistic synthetic DIA-LC-MS/MS mzML measurements derived from the real measurement to benchmark our method against an existing state-of-the-art DIA mass spectrometry analysis tool with a known exact ground-truth (Fig. 4).

In Fig. 2A, the score calculation of two interpolated measured chromatograms is demonstrated. Interpolation of both chromatograms is required, since cosine similarity requires two vectors of identical dimensions. The cosine similarity is then calculated and returns a value between 0 and 1, interpreted as a percentage: 100 % means fully identical shapes, and 0 % means fully dissimilar shapes. Figure 2B shows how the algorithm reconstructs a simulated convoluted system using Gaussian distributions as elution profiles, and Fig. 2C, E show how the algorithm’s performance on this same convoluted system reacts to pressure from increasingly challenging conditions. The predicted fragment contributions can be compared directly with the theoretical fragment contributions, since we know the ground-truth fragmentation efficiency for all precursors in a simulated example. Optimal deconvolution means that the shape of each theoretical ground-truth fragment is fully recovered from the convolved sum of all ground-truth fragments. The example deconvolution in Fig. 2B achieves an almost optimal score of 99.59 %. In Fig. 2C, on the left, the influence of the sample rate on the algorithm is visualized, and on the right, the score distributions of over 500 simulated convoluted systems with different sample rates (i.e. different amounts of data points per chromatogram) are shown. We performed similar experiments with increasingly worse signal-to-noise ratios in Fig. 2D and with increasingly high retention time overlap between the precursors in Fig. 2E. These three experiments –- high noise, low sample rate, and high overlap –- demonstrate that, under controlled conditions, the algorithm’s performance deteriorates predictably and monotonically as signal complexity increases. Initially, the score remains stable with a slight downward trend, then, as conditions worsen, the average score decreases more quickly.

As, in contrast to simulated data, a complete ground-truth is not available for measured DIA lipidomics data, we manually annotated the selected convoluted system using established lipid fragment libraries from LipidCreator (Peng *et al.* 2020). This annotation enables us to qualitatively evaluate the deconvolution algorithm’s behavior on real data by distinguishing fragments known to be associated with a given precursor from co-isolated but unrelated signals. Accordingly, the evaluation focuses on the expected qualitative outcome of a correct deconvolution, namely, whether reconstructed fragment chromatograms align coherently with the corresponding precursor elution profiles and whether reconstructed MS^2^ spectra retain signals from known fragments and show reduced interference from fragments not attributable to that precursor.

Figure 3 illustrates the deconvolution performance on real data. We selected one convoluted system and manually annotated it to demonstrate the algorithm’s core behavior on annotated cumulative and reconstructed MS^2^ spectra. Figure 3A and B shows the measured chemical space of one isolation frame in MS^1^ and the associated MS^2^ space on the retention time and *m/z* axes in 2D and 3D. We selected three precursors to demonstrate the application of the deconvolution algorithm, one without overlapping lipids during most of its elution time at *m/z* 788.5229 and retention time 44.3 min, and two from a strongly convoluted system at retention time 46.3 min and 47.5 min for *m/z* 786.5285 and 797.5321. For these three precursors, the reconstructed fragment chromatograms are shown in Fig. 3C, while Fig. 3D, E compares the cumulative fragment spectrum of all fragments within the isolation window over the precursor’s retention time with that of only the associated fragments. For each precursor, interference with the LipidCreator-annotated fragments is qualitatively reduced.

**Figure 3:**
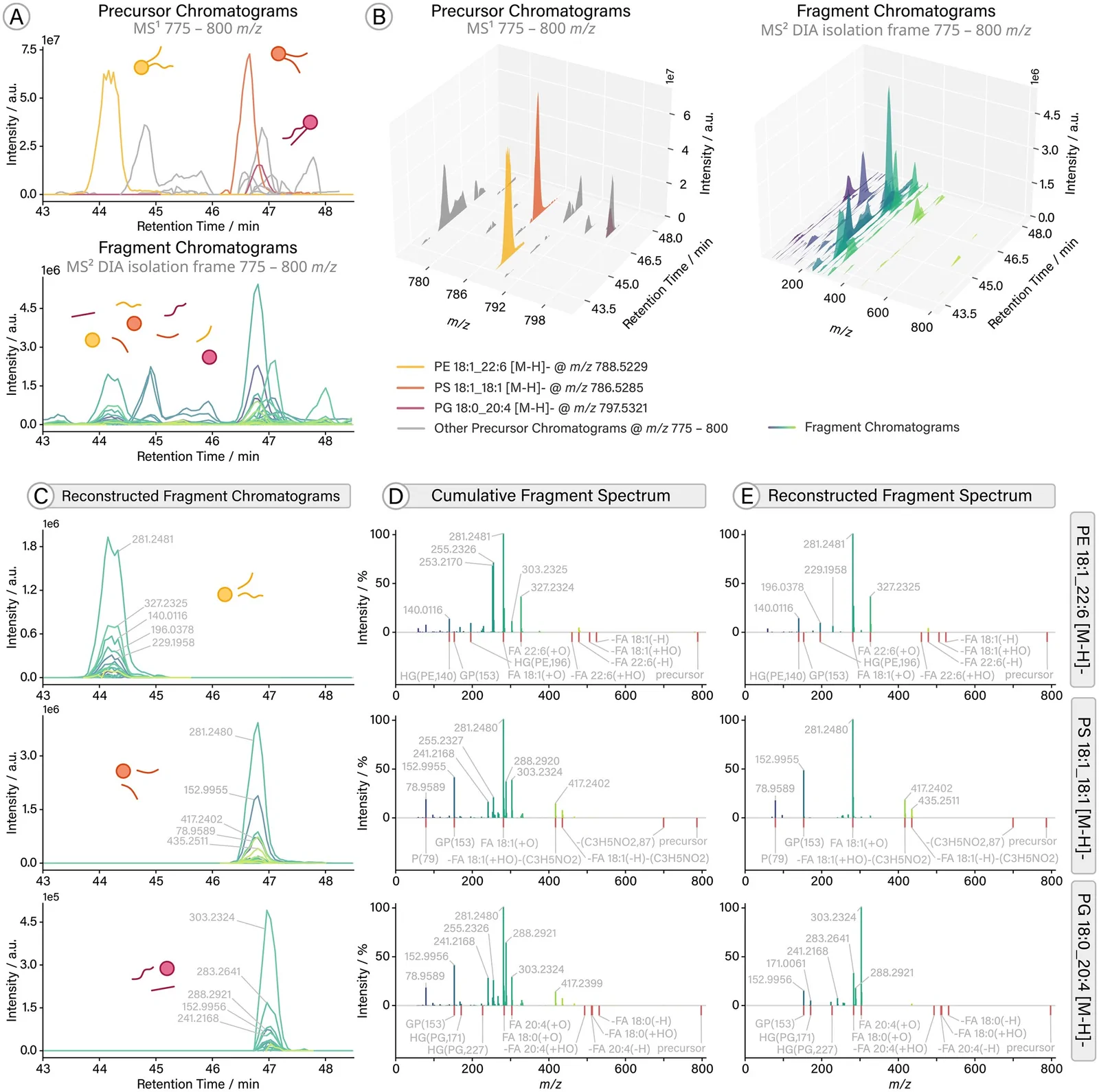
Deconvolution algorithm applied to a lipidomics DIA-LC-MS/MS experiment. (A) Region of interest around three selected precursors: PE 18:1_22:6 [M − H]-, PS 18:1_18:1 [M − H]-, and PG 18:0_20:4 [M − H]- after chromatogram extraction in the measurement space in MS1 and MS2 defined by the m/z and retention time axes. Lipid identification was manually conducted for these examples. All ions inside the shown MS1 precursor space are fragmented and together produce the signals in the shown MS2 fragment space. These chromatograms serve as input to the algorithm. (B) Region of Interest around three selected precursors in 3D. (C) Fragment chromatograms reconstructed by the algorithm and reassigned to their corresponding precursor chromatograms. (D) Cumulative MS2 spectra over the precursor elution time before applying the algorithm. (E) Final reconstructed MS2 spectra for the three precursors after deconvolution and fragment reassignment. This spectrum is the algorithm’s output.

To benchmark the mixture model deconvolution (MMD) against an established method, we compared its performance to MS-DIAL 5, using the realistically simulated dataset as described above (see Section 2). Figure 4A illustrates the comparison of the identification improvement of both methods. Here, the reconstructed MS^2^ spectra are compared to the ground-truth MS^2^ spectrum for each precursor using cosine similarity: Fig. 4A1 shows the comparison of both methods precursor-by-precursor, where points below the diagonal mean the MMD performed better and points above the diagonal mean MS-DIAL 5 performed better. Note that the majority of the points are below the diagonal close to score value 1.0, which indicates an overall better performance for the MMD. Figure 4A2 presents a histogram of cosine similarity scores across all precursors; and Fig. 4A3 splits this distribution by DIA isolation window size as violin plots. These figures again demonstrate the superior performance of MMD compared to MS-DIAL 5. Figures 4A1–3 show that the cosine similarity is consistently significantly higher over all isolation window sizes compared to the state-of-the-art method; one paired nonparametric permutation test per isolation window confirms a *P*-value *P* < 1e^−5^ for all five isolation window sizes. Especially with 50 Da isolation windows the mean cosine similarity of 289 precursors is 0.733 for MMD and 0.509 for MS-DIAL 5.

**Figure 4:**
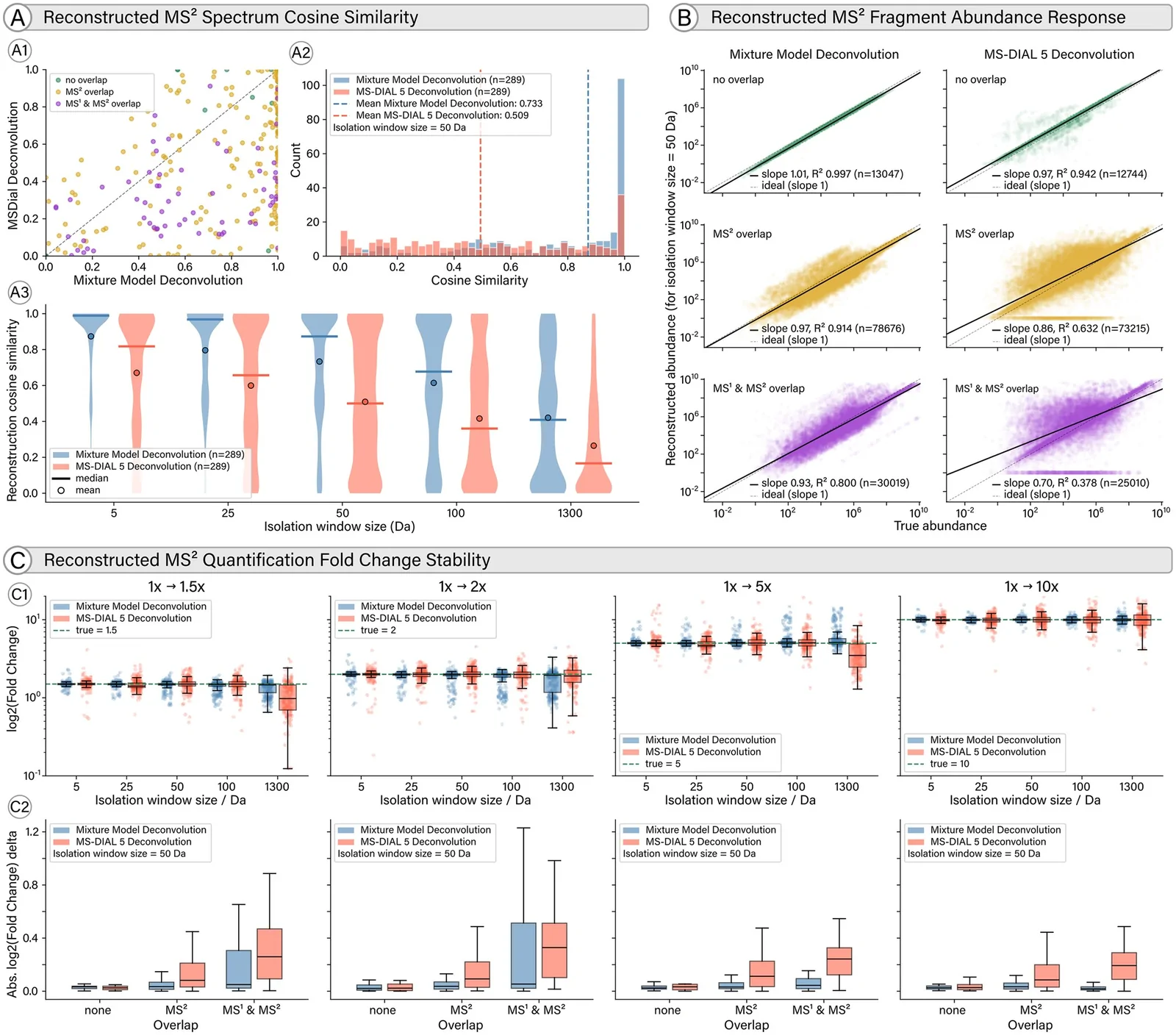
Comparison of the mixture model deconvolution (MMD) algorithm and current state-of-the-art MS-DIAL 5 DIA mass spectrometry analysis tool deconvolution. (A) Analysis of the possible identification improvement by comparing reconstructed MS2 spectra with the ground-truth MS2 spectra for each precursor using cosine similarity. (A1) Cosine similarity comparison between both approaches for each precursor in the ground-truth. Points on the diagonal line mean both methods performed equally well, points below the diagonal mean the MMD performed better, points above the diagonal mean the MS-DIAL 5 deconvolution performed better. The plot clearly shows more points on the MMD side, especially with values = 1.0. (A2) Histogram of the distribution of cosine similarities for each precursor in the ground-truth. The distributions of cosine similarities confirm the statement of A1 and also show the mean is significantly higher with MMD. (A3) Violin plot of the distributions of cosine similarities for each precursor in the ground-truth for different DIA isolation window sizes. (B) Analysis of the fragment abundance response. For each fragment in the ground-truth, the abundance is compared to the reconstructed abundance from both methods. The visualization splits all fragments in three overlap scenarios: Fragments with no overlap, “MS2 overlap” means fragments which are convoluted with at least one other precursor, and “MS1 & MS2 overlap” fragments where two or more precursors have the same m/z ratio, co-elute, and share at least one fragment. “MS1 overlap” was also included in the analysis, but there are no precursors in the dataset that just have the same m/z ratio, co-elute, and do not share any fragments. (C) Analysis of the fold change response stability using a realistic quantification approach for each precursor. For each precursor only fragments from the ground-truth are selected that occur in each measurement, which are then summed up to result in one abundance value. (C1) Comparison of the log2(Fold Change) for all precursors in the ground-truth over multiple fold change variation experiments with different DIA isolation window sizes. Boxplots closer to the green line means more precursors have been correctly quantified according to the ground-truth. (C2) Comparison of the different overlap types using their absolute log2(Fold Change) delta as described in (B) over the same fold change and DIA isolation window size variation experiments as in (C1). Both (B) and (C2) show a very similar performance on non-overlapping precursors, which is to be expected as there is no deconvolution necessary, but for both MS2 overlap and MS1 & MS2 overlap the MMD approach is able to more accurately reconstruct the ground-truth abundances.

In Fig. 4B, the fragment abundance response of both methods is compared against the ground-truth for each fragment, split by the overlap scenarios OS1 “no overlap”, OS2 “MS^2^ overlap”, OS3 “MS^1^ overlap”, and OS4 “MS^1^ & MS^2^ overlap” as defined in the Methods upon a 50 Da isolation window size; no precursors in the dataset had matching *m/z* and co-elution without also sharing a fragment, so the OS3 stayed empty. Points closer to the identity line indicate a better quantitative reconstruction. Figure 4B shows a more consistent response over all overlap scenarios for MMD compared to MS-DIAL 5. Note that most of the error is produced by the OS4 in both methods. Fitting a regression for all precursors within an overlap scenario shows, the performance is very similar on the OS1, both with an *R*^2^  >0.9. In the OS2 it shows similar performance to the OS1 for MMD with an *R*^2^ of 0.914 but a noticeable deviation from the optimal regression for MS-DIAL with an *R*^2^ of 0.632, and an even more pronounced difference in the OS4 with an *R*^2^ of 0.800 for MMD and 0.378 for MS-DIAL.

Figure 4C compares log_2_ fold change response stability using the realistic quantification approach from the Methods. Figure 4C1 compares the log_2_ fold change of all ground-truth precursors across the abundance multiplier and isolation window size variation experiments, where boxplots closer to the reference line indicate that more precursors were correctly quantified. Figure 4C2 splits this comparison by the overlap scenarios from Fig. 4B, again at an isolation window size of 50 Da. Figure 4C1-2 shows that the log_2_ fold change stays consistently closer to the ground-truth with less variance for MMD than for MS-DIAL 5, across all abundance multipliers and isolation window sizes up to AIF, where both methods deviate more strongly from the ground-truth compared to the other isolation window sizes. A paired nonparametric permutation test confirms the variance of the log_2_ fold change is significantly smaller for MMD than for MS-DIAL 5, with *P* < .001 in all cases, while the mean does not significantly differ in most cases. Testing the log_2_ fold change error instead, the difference in distribution mean is statistically significant with *P* < .001 across all isolation window sizes and abundance multipliers. The difference in distribution variance is significant with *P* < .001 for all isolation window sizes smaller than 100 Da, *P* < .01 at 100 Da, and *P* < .01 at AIF for the 1.5×, 5×, and 10× abundance multipliers. Further results on the comparison between MMD and MS-DIAL 5 are available in the Supplementary Data Section 3.

Following deconvolution, common lipid identification strategies can be applied for the reconstructed precursor-specific MS^2^ spectra. Fragments whose reconstructed chromatographic shapes deviate strongly from the corresponding precursor elution profile can be excluded from identification, as they likely originate from co-isolated lipids or other artefacts. Quantification can be performed on MS^1^ precursor chromatograms or on reconstructed MS^2^ fragment chromatograms, depending on the analytical objective.

Together, the simulation and the real lipidomics data example demonstrate that the proposed deconvolution algorithm yields precursor-specific fragment chromatograms and MS^2^ spectra with reduced chemical noise and interference from co-isolated lipids. The example illustrates the practical feasibility of the approach and its qualitative benefit under both controlled and experimental conditions. The algorithm is implemented as an open-source software library in multiple programming languages, and we hope it will be useful for integration into existing DIA-based lipidomics workflows.

## 4 Discussion

In DIA-based lipidomics data acquisition, chimeric co-eluting fragment ions complicate fragment attribution, interfere with identification, and limit the use of fragment-level quantification in LC-MS/MS workflows. To address this problem, we introduced a method that interprets fragment chromatograms as mixture models by exploiting the temporal correlation between fragment chromatograms and their respective precursor chromatograms. The proposed approach assigns fragment ions to their precursors and separates overlapping fragment signals into individual chromatograms. From these deconvoluted chromatograms, MS^2^ spectra are reconstructed in a single step, enabling subsequent lipid identification and quantification.

In the trivial case of non-overlapping precursors, the algorithm automatically assigns all matching fragments to the precursor. The algorithm is highly parallelizable as each convoluted system can be computed independently, allowing for efficient usage of resources and fast execution time on modern hardware. In practice, long tails of elution profiles often lead to large, convoluted theoretical systems, as they create minor overlaps that are trivial to separate. To reduce the complexity and computational cost of these systems, only the region surrounding the centre of each elution peak is considered when defining a convoluted system, while long, low-intensity tails are excluded.

Under controlled conditions where the ground-truth is known, the algorithm reliably recovers the correct solution, as demonstrated by the simulation results in Fig. 2. As signal complexity increases, higher noise levels, reduced sample rate, or increased precursor overlap do not linearly affect the scores. Scores remain high, close to 100 %, followed by a sharp drop-off near the complexity limit. Stable performance is achieved if a system has at least 5 data points per chromatogram, a signal-to-noise ratio above 1, and a maximum overlap between all precursors of 60 %. A low score is an indicator that even though the system could be solved mathematically, the resulting chromatograms might not be suited for quantification or confident identification.

In practice, this predictable score drop-off is primarily driven by the biological sample or by the acquisition method design. If the sample rate is too low, i.e. there are too few data points per chromatogram, peak shapes are no longer suitable for quantification. If the MS^2^ sample rate is too low relative to the MS^1^ sample rate, peaks may be missed if they are not detected in MS^2^. The other way around, if there are too few MS^1^ scans relative to MS^2^, the sample rate of the MS^2^ scans is reduced accordingly due to interpolation, which may result in misshapen or skipped MS^2^ peaks. If the noise behaved symmetrically in MS^1^ and MS^2^, it would be no issue, but it is independent of whether the mass spectrometer measures precursors or fragments (Urban and Štys 2015) and therefore distorts the chromatogram shapes. If isotopes overlap with the isolation window start or end, the isotope pattern in MS^2^ is incomplete, and the MS^2^ peaks may be removed as artifacts. If the isolation frame ends because the isolation window sequence changes during the elution of a precursor, the fragment chromatograms are cut off and continued in a different isolation frame. Both halves of the fragment chromatogram still align with the precursor elution profile, but the quantification may be impacted and the score calculation penalizes the cut-off shape of both fragment chromatograms. One possible solution is to optimize the analytical method for less isolation window sequence changes.

Apart from these practical constraints, there are fundamental limits to separability. If two or more precursors share a fragment in the same isolation window, elute at the same time, and have the same peak shape, the algorithm’s theoretical limit is reached, meaning their shared fragment can no longer be separated. In this case, the algorithm produces proportional fragment assignments to the precursor intensity. For example, if two ions have a ratio of, e.g. 3:1, any fragment with the same elution profile will be split according to this ratio, no matter if it solely originates from one ion or both. In this case, the reconstructed deconvoluted MS^2^ chromatograms may be used for identification, but due to the described limitation, they cannot be used for reliable quantification. When fragment chromatograms are successfully deconvoluted, the resulting precursor-specific fragment spectra allow straightforward integration into established identification and quantification pipelines with only minor adjustments, as they resemble DDA data, just with one MS^2^ spectrum of all associated fragments. The results demonstrate effective application on real datasets (Fig. 3). On a realistic simulated benchmark (Fig. 4), the method provides a significantly better foundation than MS-DIAL 5, with higher cosine similarity between reconstructed and ground-truth MS^2^ spectra and significantly more accurate and stable chromatogram integration for subsequent identification and quantification. Further comprehensive quantitative assessment will require a measured benchmark dataset of a strongly convolved lipid set with fully annotated MS^1^ and MS^2^ signals as a gold standard. An evaluation of the effect of deconvolution on lipid identification performance is the subject of ongoing work. The results on simulated and manually annotated measured data show this approach is statistically sound and feasible in practice.

Beyond algorithmic improvements, the practical performance of the deconvolution output primarily depends on the quality of the data and, consequently, on the underlying acquisition method. Analytical chemists can optimize their methods for the deconvolution approach by selecting isolation window widths that balance two opposing effects: wider window sizes reduce the total number of windows per cycle, shortening the cycle time and thereby increasing the number of MS^1^ and MS^2^ data points for each chromatogram, which improves peak shape fitting, however, wider windows also increase the number of co-isolated precursors per window, which reduces deconvolution performance. Figure 4 shows reliable operation up to 50 Da isolation windows, but this depends on the chromatographic separation, number of co-isolated precursors, chemical background matrix, and measurement noise.

A possible future extension is ion mobility spectrometry (IMS) for preseparation, as the algorithm is agnostic to the preseparation method. It can also be adopted for multidimensional separation (e.g. LC × LC or LC × IMS), where deconvolution would operate on the multidimensional space rather than the single separation dimensions. A natural direction for future work is the MS^1^-level signal separation, where isomeric and isobaric overlap is a persistent challenge in lipidomics (Kopczynski *et al.* 2024). As the comparison data in Fig. 4 show, MS^1^ deconvolution of isomeric and isobaric lipids is needed to address the “MS^1^ & MS^2^ overlap” scenario; however, deconvoluting both dimensions at once may be risky, as it can splinter chromatograms rather than resolve them. Additionally, the inference of missing MS^1^ chromatograms from their corresponding unassigned MS^2^ chromatograms present another possible extension of the method. Another direction for future work is to extend the algorithm to handle staggered and overlapping DIA windows.

## 5 Conclusion

DIA is a technique commonly used in proteomics to increase molecular coverage and improve reproducibility across measurements. These advantages also make DIA attractive for lipidomics, where it can enhance MS and MS/MS coverage and reproducibility, especially important in discovery-driven and exploratory studies. However, methodological development and dedicated software support for DIA in lipidomics remain limited, largely due to the increased complexity of chimeric MS^2^ spectra.

This work demonstrates that precursor-specific fragment spectra can be sufficiently extracted from DIA measurements using a shape-agnostic mixture model. By deconvoluting shared fragment chromatograms and assigning them to their respective precursors, false identifications will be reduced and quantification will be improved. An openly available implementation is provided to enable reproducible application and further future validation of the approach.

While challenges remain when applying DIA in lipidomics, this approach marks a significant step toward establishing DIA as a robust and reliable acquisition strategy for quantitative lipidomics. By improving fragment attribution in the presence of co-elution and shared fragmentation, DIA chromatogram deconvolution enables more robust lipid identification and more accurate quantification. This ultimately leads to more reliable downstream statistical analyses and biological interpretation.

## Supplementary Material

btag695_Supplementary_Data

## Data Availability

Source code of the software library: GitLab (Apache 2.0 License): https://gitlab.com/computational-multiomics/projects/mixture-model-deconvolution. An archived snapshot of the code that performed the experiments presented in the manuscript is available in Zenodo (Apache 2.0 License): https://doi.org/10.5281/zenodo.21218594. We developed, tested, and ran all of the simulations of our Python, C++, and Rust implementations on a Fedora 43 system with an Intel Core i7-13700T (16 Cores at 4.90 GHz, 8 Cores at 3.60 GHz) and 32 GB RAM. The MS-DIAL 5 application was run on a Windows 11 system with an AMD Threadripper 3960X (24 Cores at 3.80 GHz) and 160 GB RAM. The reference measurement and all simulated data is available in Zenodo (Apache 2.0 License): https://doi.org/10.5281/zenodo.21218594. All other data used in the manuscript is publicly available from the cited articles.
