# Supplementary material for: Improving lipid identification and quantification: chromatogram deconvolution for LC-MS/MS workflows
Source: Bioinformatics. 2026 Sep 18;42(10):btag695. doi: 10.1093/bioinformatics/btag695 (PMC13637620; doi:10.1093/bioinformatics/btag695)
Supplement: Supplementary file 1 [file btag695_supplementary_data.pdf]

Supplementary Data

1 Further Insights on Methodological Choices

1.1 Choice of Linear Interpolation

Since MS^1 and MS^2 spectra are not recorded simultaneously, precursor chromatograms must be resampled onto the retention time points of the associated fragment chromatogram before the mixture model can be evaluated pointwise; linear interpolation was chosen because it introduces no assumption about the underlying peak shape beyond local continuity. Other approaches such as higher-order interpolation or some curve smoothing algorithms might offer better performance, but need extensive testing to ensure that they do not suppress peaks or otherwise change the shape of the chromatograms too much, as this shape is the basis for the mixture model deconvolution.

1.2 Handling of Noise

Noise is not explicitly filtered or modeled prior to deconvolution. Instead, any signal in the fragment chromatogram that cannot be explained by a linear combination of the candidate precursor chromatograms is absorbed by the background weight w_0. Therefore, noise manifests as intensity assigned to this null component rather than distorting the weights w_i assigned to genuine precursors. In the convoluted system simulation (Figure 2), noise is added independently to the precursor and theoretical fragment chromatograms prior to convolution, to simulate independent background noise in the measurement.

1.3 EM Initialization

Each weight $w_i^{(0)}$ is initialized proportional to the integral of its corresponding precursor chromatogram, reflecting the assumption that a precursor's own abundance is a reasonable prior for its expected contribution to the observed fragment curve. This reduces the number of iterations to convergence and lowers the risk of the algorithm settling on a local rather than the global maximum. An alternative assumption would be to distribute the initial weights uniformly over all precursors or to heuristically precompute the factors.

1.4 Choice of Cosine Similarity

Cosine similarity was chosen for the shape score $s_s(\vec{a}, \vec{b})$ because it depends only on the relative shape of two chromatograms and is invariant to absolute intensity scale. This is required because precursors and their fragments ionize and fragment with different, unknown efficiencies, so their absolute intensities are not directly comparable. Since shape similarity alone does not capture correct scaling, the weight score $s_w(\vec{a}, \vec{b})$ is combined with it in $s_c(\vec{a}, \vec{b})$ to jointly evaluate shape and magnitude.

1.5 Overlap Ratio for Simulated Convoluted Systems

To quantify how strongly the precursor elution profiles overlap in a simulated convoluted system, we define an overlap ratio r_{O} (Equation S3), used for the overlap annotation in Figure 2E. We define the overlap score on a set of k curves from n y-values on a shared grid of n x-values, where $\vec{x} = (x_1, ..., x_n)$ with $x_0 < ... < x_n$ and $\vec{y}_i = (y_{i,1}, ..., y_{i,n})$ with $i \in \{1, ..., k\}$. This fits the layout of k fragment chromatograms with intensities measured over n spectra, meaning all chromatograms are already n-dimensional and no interpolation is necessary. The idea is to subtract the integral of the maximum of all k fragment chromatograms from the integral of the measured fragment chromatogram and divide this difference by the integral of the measured fragment chromatogram. For the integral, we use the chained trapezoidal rule again.

Let the sum $\vec{u}$ (Equation S1) and the maximum $\vec{v}$ (Equation S2) be vectors on the same grid of $j \in \{1, ..., k\}$ x-values:

$$\vec{u} = (u_1, ..., u_n) = (\sum_i y_{i,1}, ..., \sum_i y_{i,n}) \tag{S1}$$

$$\vec{v} = (v_1, ..., v_n) = (\max_i(y_{i,1}), ..., \max_i(y_{i,n})) \tag{S2}$$

The overlap between the k curves (r_{o}, Equation S3) is then defined as:

$$r_{\text{O}}(\vec{x}, \vec{u}, \vec{v}) = \frac{\sum_{j=0}^{n-1} \frac{x_{j+1} - x_j}{2}((u_j - v_j) + (u_{j+1} - v_{j+1}))}{\sum_{j=0}^{n-1} \frac{x_{j+1} - x_j}{2}(u_j + u_{j+1})} \tag{S3}$$

We obtain a measure of how much overlap there is in a convoluted system (r_{O}, Equation S3): if two curves do not overlap, the ratio is 0 %, if two curves overlap exactly, the ratio is 100 %, if three curves overlap exactly, the ratio is 200 %.

Because $r_{\text{O}}(\vec{x}, \vec{u}, \vec{v})$ is computed from integrated curves, it reflects only the aggregate degree of overlap within a convoluted system and not the number of precursors involved or where along the retention time axis the overlap occurs; a system with one strongly overlapping pair and a system

with several weakly overlapping curves can therefore yield similar values. It is also insensitive to local structure, since a small overlap concentrated at peak apex is not distinguished from the same overlap spread across peak shoulders, even though these cases pose different levels of difficulty for the deconvolution.

1.6 Statistical Tests

We used paired nonparametric permutation tests for:

- the difference in cosine similarity distribution mean.
- the difference in $\log_2$ fold change distribution mean.
- the difference in $\log_2$ fold change distribution variance.
- the difference in $\log_2$ fold change error distribution mean.
- the difference in $\log_2$ fold change error distribution variance.

We tested whether Mixture Model Deconvolution and MS-DIAL differ significantly in quantification accuracy and precision, specifically in the mean cosine similarity score, the mean and spread of $\log_2$ fold change, and the mean and spread of $\log_2$ fold change error, at each isolation window and target fold change. Since the same precursor is quantified independently by both methods, each comparison is paired, and we test the null hypothesis that the paired values are exchangeable between methods using paired sign-flip permutation tests, avoiding any assumption on the shape of the underlying distributions.

This choice rules out several standard alternatives. A Welch's t-test and the Wilcoxon rank-sum test assume independent samples and would discard the pairing, testing a weaker hypothesis than the one of interest. A paired t-test requires the paired differences to be approximately normally distributed, and the Wilcoxon signed-rank test requires them to be symmetric around the median; neither assumption holds reliably across our data, as cosine similarity scores are bounded in [0,1] and often skewed toward the extremes, and $\log_2$ fold-change errors are heavy-tailed and occasionally bimodal. Paired sign-flip permutation tests make no assumption on the shape, symmetry, or homoscedasticity of the underlying distributions, only that each pair's two values are exchangeable under the null hypothesis. Concretely, each pair's values are randomly and independently reassigned between the two methods to build an empirical null distribution of the test statistic (implemented with `scipy.stats.permutation_test(permutation_type='samples')`), computed exactly by enumerating all 2^n sign patterns for $n \geq 20$, and by Monte Carlo resampling (10^5 permutations) otherwise.

For the difference in cosine similarity, $\log_2$ fold change, and $\log_2$ fold change error, the mean was used as the test statistic. For the difference in spread of $\log_2$ fold change, and of $\log_2$ fold change error between methods, the difference in sample variances was used instead, applying the same paired sign-flip procedure rather than Levene's test, since Levene's test assumes independent

samples and relies on an F-distribution approximation to its null that we preferred not to invoke given the non-normality of our data.

1.7 Manual Dataset Annotation

The scope of the presented method is data reduction and feature extraction, not lipid identification; the algorithm deconvolutes chimeric MS^2 chromatograms and reconstructs precursor-fragment relationships, but does not assign lipid identities to precursors or fragments. The deconvoluted fragment chromatograms are closer to the ground truth than the raw, convoluted signal, as shown by the reduction in false annotations and quantification error in Figure 4, so any downstream annotation step therefore operates on input closer to the ground truth than it would without deconvolution, whether that step is manual or automated. Annotation of the measured dataset presented here was performed manually because no identification pipeline suited to deconvoluted DIA lipidomics data was available to us at the time, making manual annotation the only route to a verified benchmark reference set.

LipidXplorer is open-source software (GNU GPL v2) that supports the quantitative characterization of complex lipidomes by interpreting large datasets of shotgun mass spectra, restricted to direct-infusion data without a chromatographic dimension, so it cannot be applied to LC-DIA fragment chromatograms without modification. The open-source software LDA2 (GNU GPL v3) covers LC-coupled data, but only for DDA acquisition, and so does not address the co-isolation and co-fragmentation problem this work targets. MS-DIAL is open-source software (GNU LGPL v3) for untargeted metabolomics and lipidomics data that supports LC-DIA acquisition, and its automatic annotation is the closest existing match to a DIA lipidomics identification tool. In our hands, however, its reference library proved too limited so we had to develop our own lipid fragment database for the annotation. Furthermore, convoluted systems frequently caused wrong annotations, and rigorous filtering and manual intervention was so often necessary, forcing us to redo the annotation process manually. This limitation was the original motivation for developing the deconvolution algorithm presented here.

No open-source tool currently performs automated lipid identification on deconvoluted DIA-LC-MS/MS fragment chromatograms with a library broad enough to resolve co-eluting, co-fragmenting species; closing this gap requires either extending an existing DDA- or shotgun-oriented annotation library to accept deconvoluted DIA fragment chromatograms in MGF format, or building a dedicated DIA lipid identification engine that consumes the deconvolution output directly. We are not aware of an identification pipeline for lipidomics that can consume MGF data from an external deconvolution step, but nothing about the format itself prevents this, since it is already common on the output side of such tools. Either would complete an end-to-end pipeline from raw DIA acquisition to annotated, quantified lipid species without a manual annotation step.

2 Runtime Benchmark

We benchmarked the deconvolution algorithm on simulated convoluted systems as described in the methods section of the paper. Its memory and runtime requirements are both in the range of $O(kn)$, where k is the number of possible precursors for a fragment chromatogram, and n is the number of data points in the fragment chromatogram. For a real measurement, a realistic range for the number of precursors is from 1 to 100 for regular DIA and up to 1000 for AIF, and the amount of data points per chromatogram could range between 5 and 50. Figure S1A shows the runtime of the Python implementation of the algorithm as described in the paper. We fit an exponent b to the formula $y = an^b + c$, which resulted in a $b = 1.00 \pm 0.00$ with a Pearson R^2 of 1.000, and performed a linear fit to the formula $y = an + c$, which resulted in an R2 of 1.000, proving an exactly linear runtime in relation to the number of precursors. Figure S1B shows the isolation of a possible logarithmic component, which was not evident, as the slope of the linear fit is not positive.

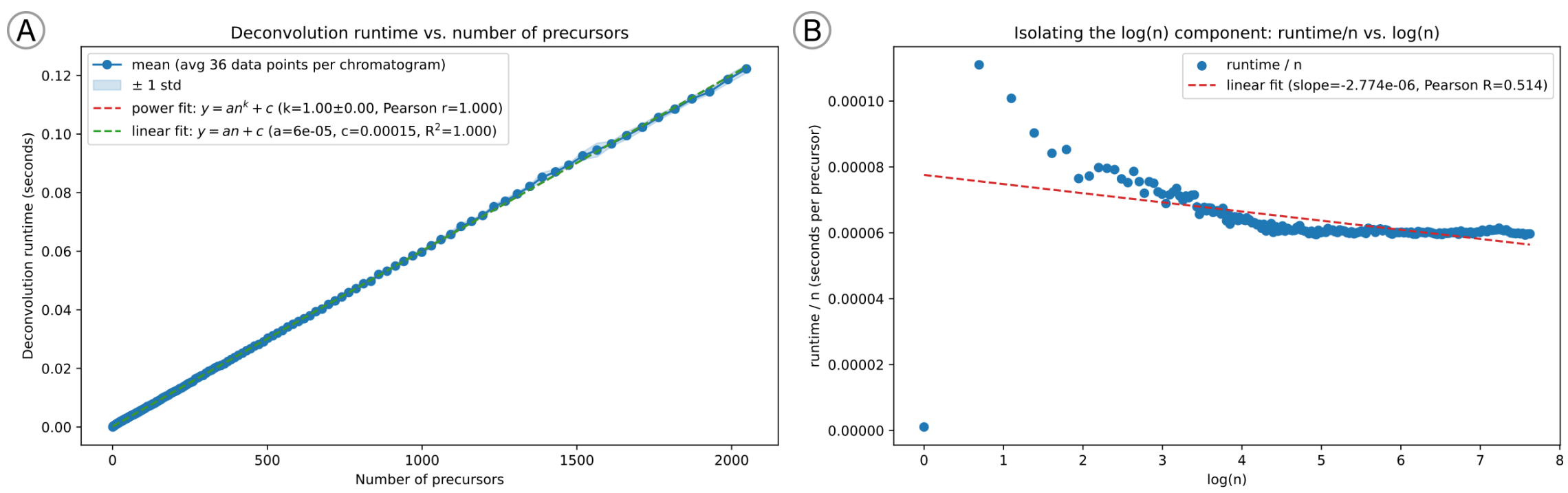

Figure S1: Runtime benchmark of the Python implementation of the algorithm as described in the paper on simulated convoluted systems with a varying number of precursors. **A**) Runtime of the algorithm compared to the number of precursors with a power fit and a linear fit calculated for the data points. The runtime is linear in relation to the number of precursors. **B**) Isolation of a possible logarithmic component in the runtime. A positive slope of the linear fit would show evidence for such a logarithmic component, which is not evident here.

We implemented a vectorized version of the algorithm in Python and NumPy, with which we were able to improve the runtime by a factor of 10, as shown in Figure S2, analogous to Figure S1. For real measurement files the practical runtime is mainly dependent on the chromatographic separation, the isolation window size, gradient length, sampling frequency, and the chromatogram extraction and preprocessing quality. On the measured dataset (25 Da isolation window size, average of 22.16 data points per fragment chromatogram) with isotope correction and singleton deletion (9026 precursor chromatograms, 47 329 fragment chromatograms) the deconvolution took about 3.8 seconds on the hardware listed in the Code Availability Statement, however without any preprocessing and isotope correction (76 335 precursor chromatograms, 47 329 fragment chromatograms, roughly 8.5x as many precursor chromatograms but not equally distributed over all

isolation frames) deconvolution took about 15 seconds (4x runtime). For multiple measurement files, the algorithm scales linearly, as it only runs on one file at a time.

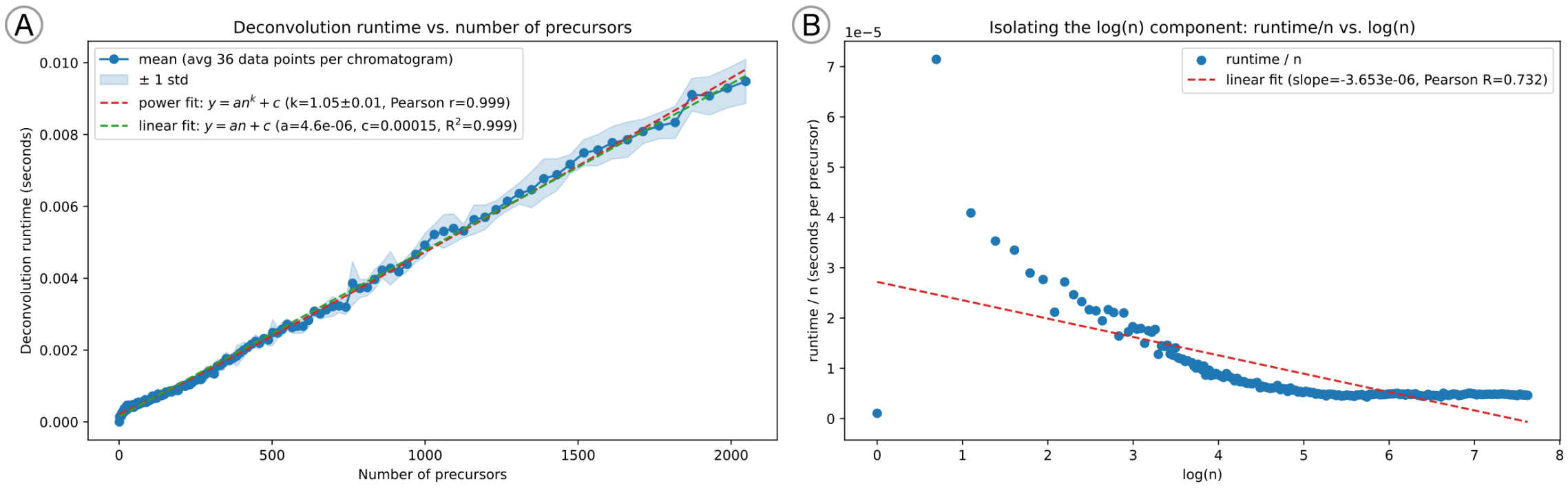

Figure S2: Runtime benchmark of the vectorized algorithm implementation in Python and NumPy with a varying number of precursors. **A**) Runtime of the algorithm compared to the number of precursors with a power fit and a linear fit calculated for the data points. The runtime is linear in relation to the number of precursors. **B**) Isolation of a possible logarithmic component in the runtime. A positive slope of the linear fit would show evidence for such a logarithmic component, which is not evident here.

3 Further Method Comparison Figures and Data

3.1 Simulated Data

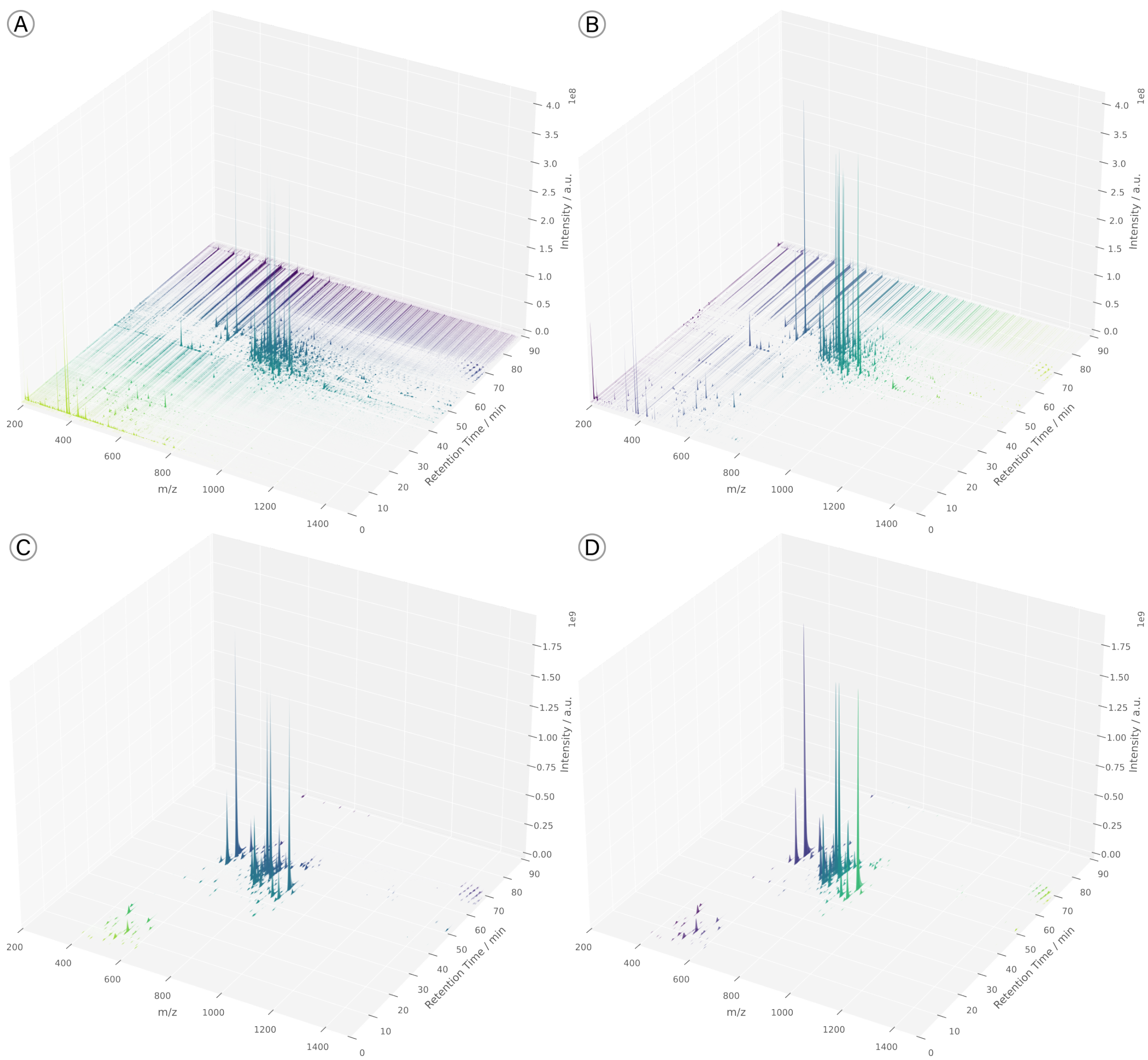

Figure S3: Visualization of MS^1 spectra and chromatograms extracted from the measured and simulated datasets. The simulated dataset is created from only the annotations from the measured dataset. **A**) Measured dataset MS^1 spectra. **B**) Measured Dataset MS^1 chromatograms. **C**) Simulated dataset MS^1 spectra. **D**) Measured Dataset MS^1 chromatograms.

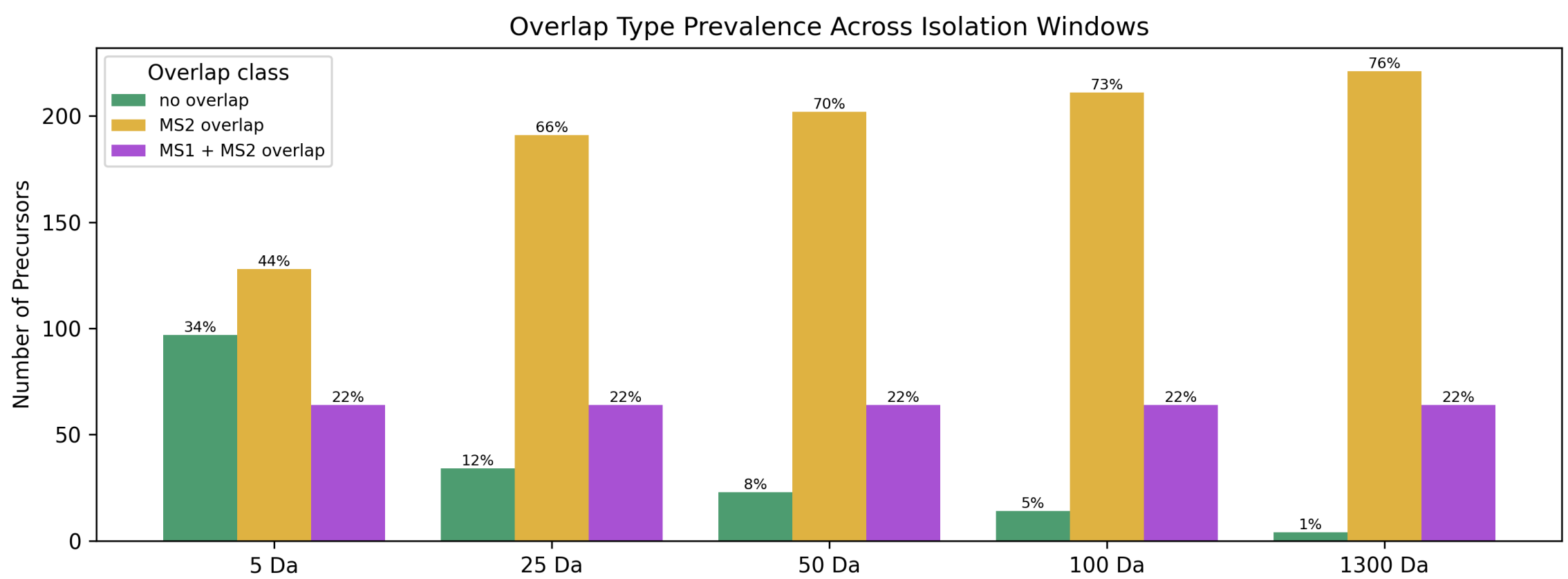

Figure S4: Overlap type prevalence across isolation windows. The visualization splits all fragments in three overlap scenarios: Fragments with no overlap (OS1), "MS^2 overlap" (OS2) means fragments which are convoluted with at least one other precursor, and "MS^1 & MS^2 overlap" (OS4) fragments where two or more precursors have the same m/z ratio, co-elute, and share at least one fragment. "MS^1 overlap" (OS3) was also included in the analysis, but there are no precursors in the dataset that just have the same m/z ratio, co-elute, and do not share any fragments. Precursors with MS^1 overlap do not change, as the size of the isolation window has no influence on if they are co-isolated or not.

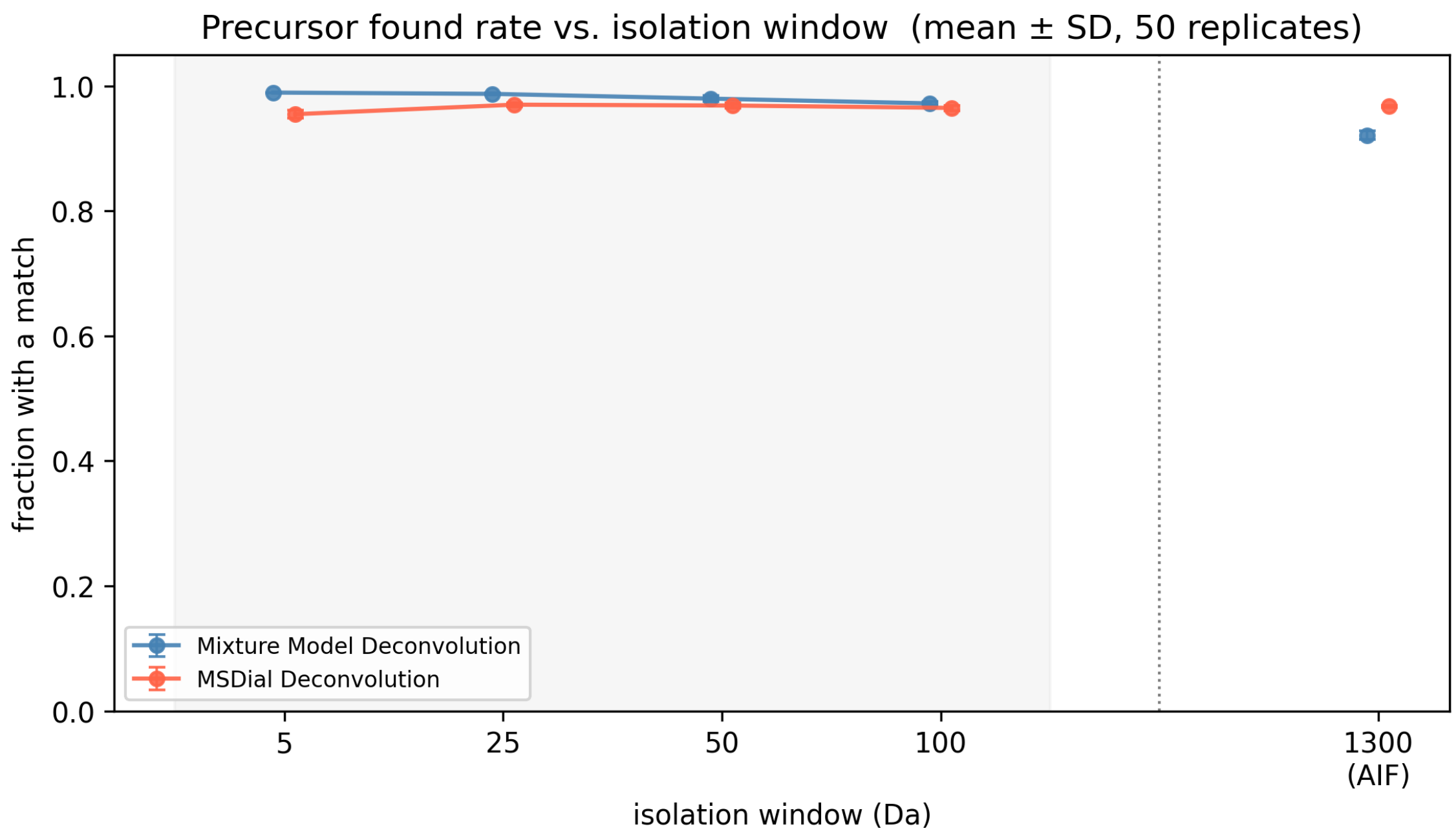

Figure S5: Percentage of found precursors over different isolation window sizes. Both approaches recover > 90 % of all precursors in the ground truth from the measurement in all cases. This only evaluates the chromatogram extraction and feature detection workflow, not the deconvolution methods, but it is an essential quality assurance measure to interpret the deconvolution results.

3.2 Reconstructed MS^2 Spectrum Cosine Similarity

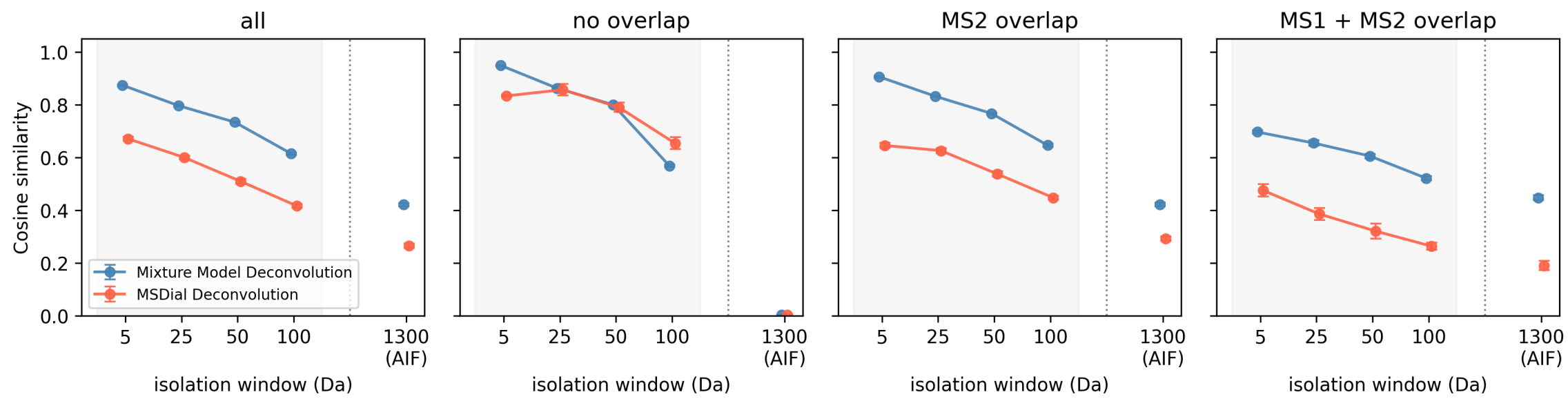

Figure S6: Cosine similarity between reconstructed MS^2 spectra and the ground-truth MS^2 spectra for both methods separated by overlap scenario: fragments with no overlap (OS1), "MS^2 overlap" (OS2) means fragments which are convoluted with at least one other precursor, and "MS^1 & MS^2 overlap" (OS4) fragments where two or more precursors have the same m/z ratio, co-elute, and share at least one fragment. "MS^1 overlap" (OS3) was also included in the analysis, but there are no precursors in the dataset that just have the same m/z ratio, co-elute, and do not share any fragments.

Isolation window size / Da	Mean difference	p-value	Statistically significant
5	0.2031	2e-5	true
25	0.1965	2e-5	true
50	0.2239	2e-5	true
100	0.1982	2e-5	true
1300 (AIF)	0.1558	2e-5	true

Table S1: Cosine similarity statistical significance analysis using a paired nonparametric permutation test. Note that 2e-5 is the minimal p-value this test produces with 10^5 resamples.

3.3 Reconstructed MS^2 Fragment Abundance Response

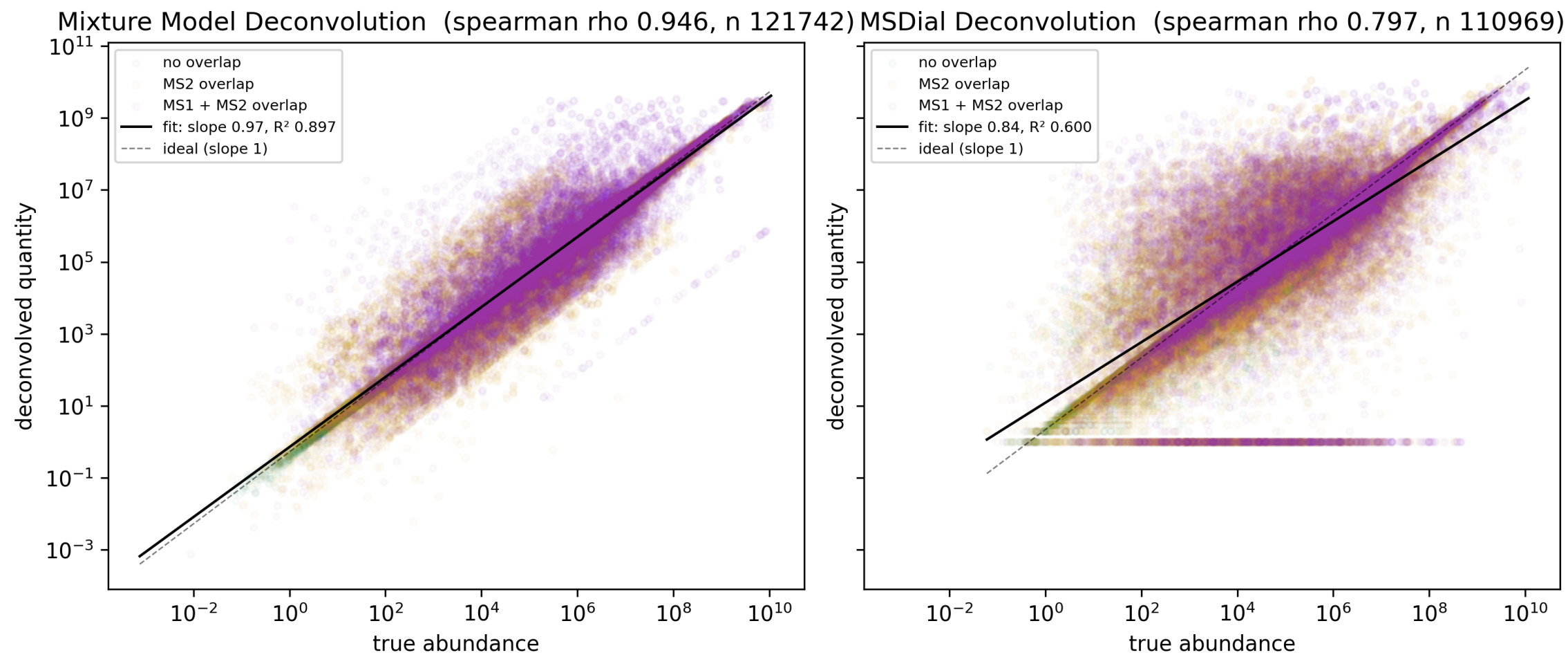

Figure S7: Fragment Abundance response comparison analogous to Figure 4B with one regression for all fragment responses. The visualization splits all fragments in three overlap scenarios: Fragments with no overlap (OS1), "MS^2 overlap" (OS2) means fragments which are convoluted with at least one other precursor, and "MS^1 & MS^2 overlap" (OS4) fragments where two or more precursors have the same m/z ratio, co-elute, and share at least one fragment. "MS^1 overlap" (OS3) was also included in the analysis, but there are no precursors in the dataset that just have the same m/z ratio, co-elute, and do not share any fragments.

3.4 Reconstructed MS^2 Quantification Fold Change Stability

$\log_2$(fc) target	Isolation window size / Da	p-value $\log_2$(fc) mean	p-value $\log_2$(fc) variance	p-value $\log_2$(fc) error mean	p-value $\log_2$(fc) error variance
1.5	5	0.5735	2e-5	2e-5	0.0002
1.5	25	0.001	2e-5	2e-5	2e-5
1.5	50	0.7323	2e-5	2e-5	2e-5
1.5	100	0.7892	2e-5	2e-5	2e-5
1.5	1300 (AIF)	2e-5	2e-5	2e-5	2e-5
2	5	0.0670	2e-5	2e-5	4e-5
2	25	0.0909	2e-5	2e-5	2e-5
2	50	0.8688	2e-5	2e-5	2e-5
2	100	0.5988	2e-5	2e-5	0.0032
2	1300 (AIF)	0.6385	0.0018	2e-5	0.1810
5	5	0.0743	2e-5	2e-5	2e-5
5	25	0.0130	2e-5	2e-5	0.0005
5	50	0.1255	2e-5	2e-5	2e-5
5	100	0.8741	0.0009	2e-5	0.0008
5	1300 (AIF)	2e-5	4e-5	2e-5	2e-5
10	5	0.3697	2e-5	2e-5	2e-5
10	25	0.1195	2e-5	2e-5	2e-5
10	50	0.7760	2e-5	2e-5	2e-5
10	100	0.3602	2e-5	2e-5	2e-5
10	1300 (AIF)	0.3528	2e-5	2e-5	0.0013

Table S2: Fold Change (fc) analysis statistical significance analysis based on fold change target and isolation window size using a paired nonparametric permutation test. Note that 2e-5 is the minimal p-value this test produces with 10^5 resamples.

$\log_2$(fc) target	Isolation window size / Da	Overlap scenario	p-value $\log_2$(fc) mean	p-value $\log_2$(fc) variance
1.5	50	none	0.2375	0.3731
1.5	50	MS^2	0.3105	2e-5
1.5	50	MS^1 & MS^2	0.6532	2e-5
2	50	none	0.4199	0.1897
2	50	MS^2	0.4450	2e-5
2	50	MS^1 & MS^2	0.6664	2e-5
5	50	none	0.5940	0.2155
5	50	MS^2	0.9992	2e-5
5	50	MS^1 & MS^2	0.0050	2e-5
10	50	none	0.8151	0.1769
10	50	MS^2	0.8185	2e-5
10	50	MS^1 & MS^2	0.8080	2e-5

Table S3: Fold Change (fc) analysis statistical significance analysis based on fold change target and overlap scenario with a fixed 50 Da isolation window size using a paired nonparametric permutation test. Note that 2e-5 is the minimal p-value this test produces with 10^5 resamples. The overlap scenarios are defined as follows: fragments with no overlap (OS1), "MS^2 overlap" (OS2) means fragments which are convoluted with at least one other precursor, and "MS^1 & MS^2 overlap" (OS4) fragments where two or more precursors have the same m/z ratio, co-elute, and share at least one fragment. "MS^1 overlap" (OS3) was also included in the analysis, but there are no precursors in the dataset that just have the same m/z ratio, co-elute, and do not share any fragments.

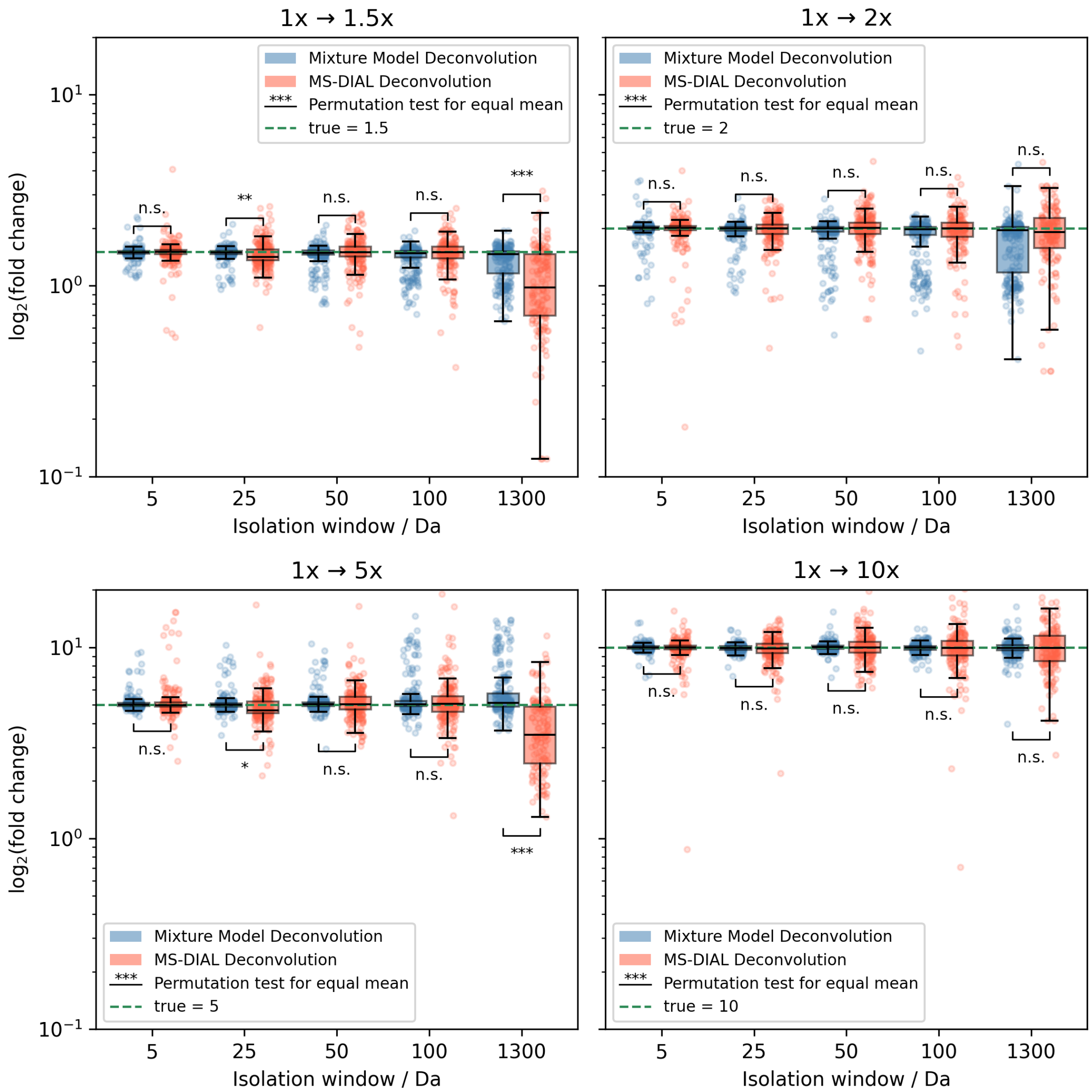

Figure S8: Fold change stability comparison between both methods for a set of fold change and isolation window size variations as described in the methods. Statistical permutation results for the **$\log_2$ fold change** distribution difference in **mean** are annotated. '***' means $p < 0.001$, '**' means $p < 0.01$, '*' means $p < 0.05$, and n.s. stands for not statistically significant.

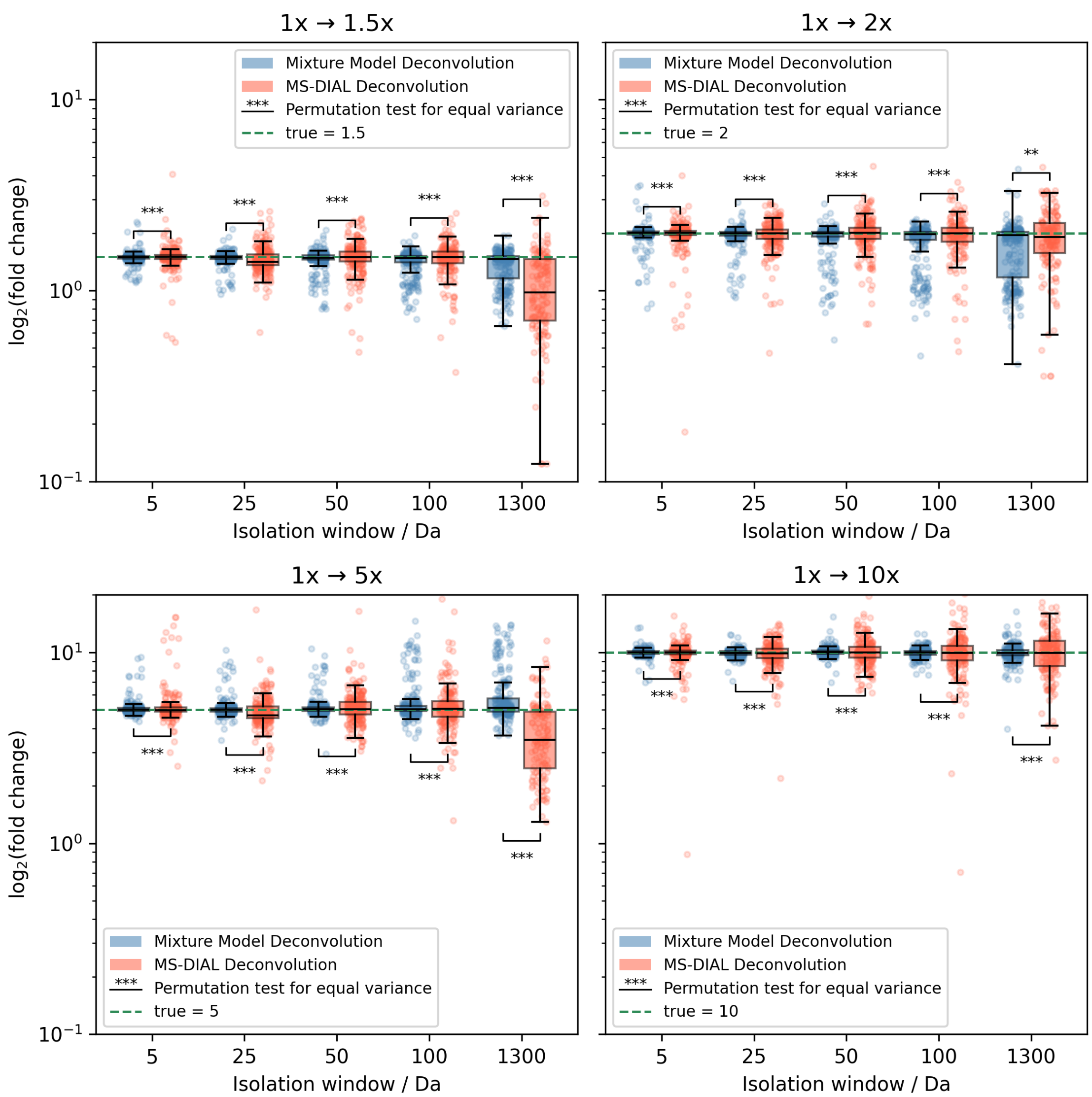

Figure S9: Fold change stability comparison between both methods for a set of fold change and isolation window size variations as described in the methods. Statistical permutation results for the **log$_2$ fold change** distribution difference in **variance** are annotated. '***' means p < 0.001, '**' means p < 0.01, '*' means p < 0.05, and n.s. stands for not statistically significant.

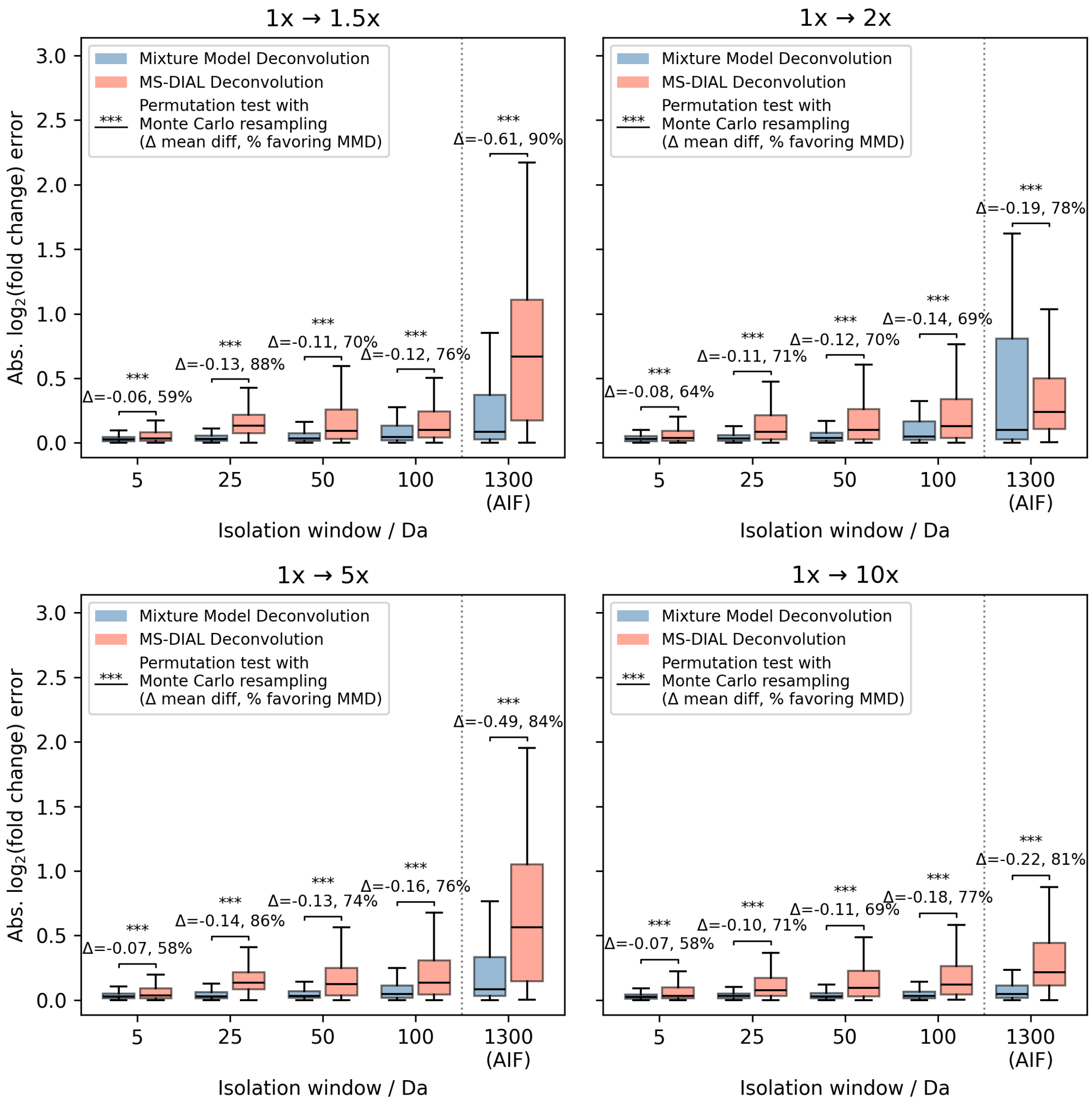

Figure S10: Fold change stability comparison between both methods for a set of fold change and isolation window size variations as described in the methods. Statistical permutation results for the **$\log_2$ fold change error** distribution difference in **mean** are annotated. '***' means $p < 0.001$, '**' means $p < 0.01$, '*' means $p < 0.05$, and n.s. stands for not statistically significant.

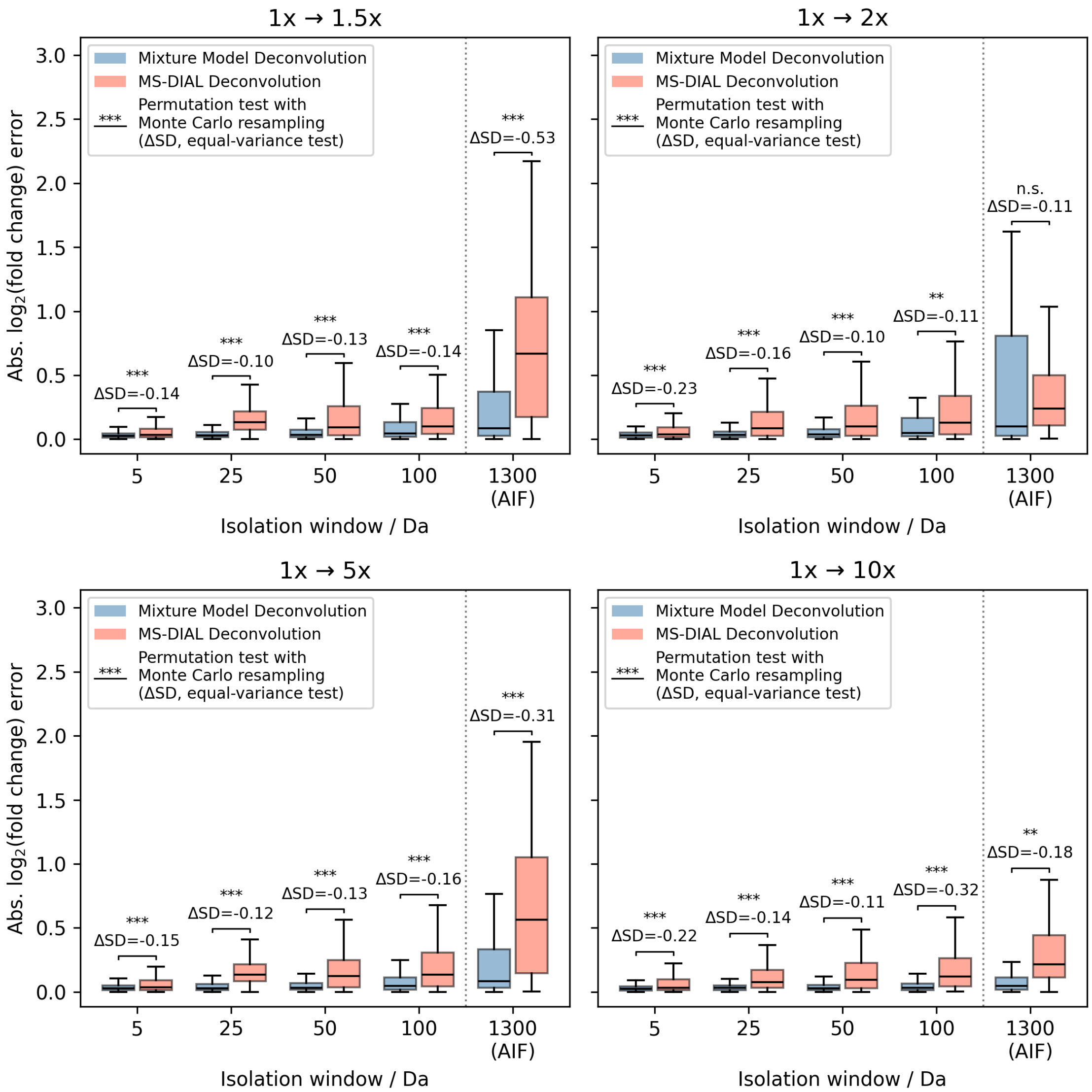

Figure S11: Fold change stability comparison between both methods for a set of fold change and isolation window size variations as described in the methods. Statistical permutation results for the **$\log_2$ fold change error** distribution difference in **variance** are annotated. '***' means $p < 0.001$, '**' means $p < 0.01$, '*' means $p < 0.05$, and n.s. stands for not statistically significant.

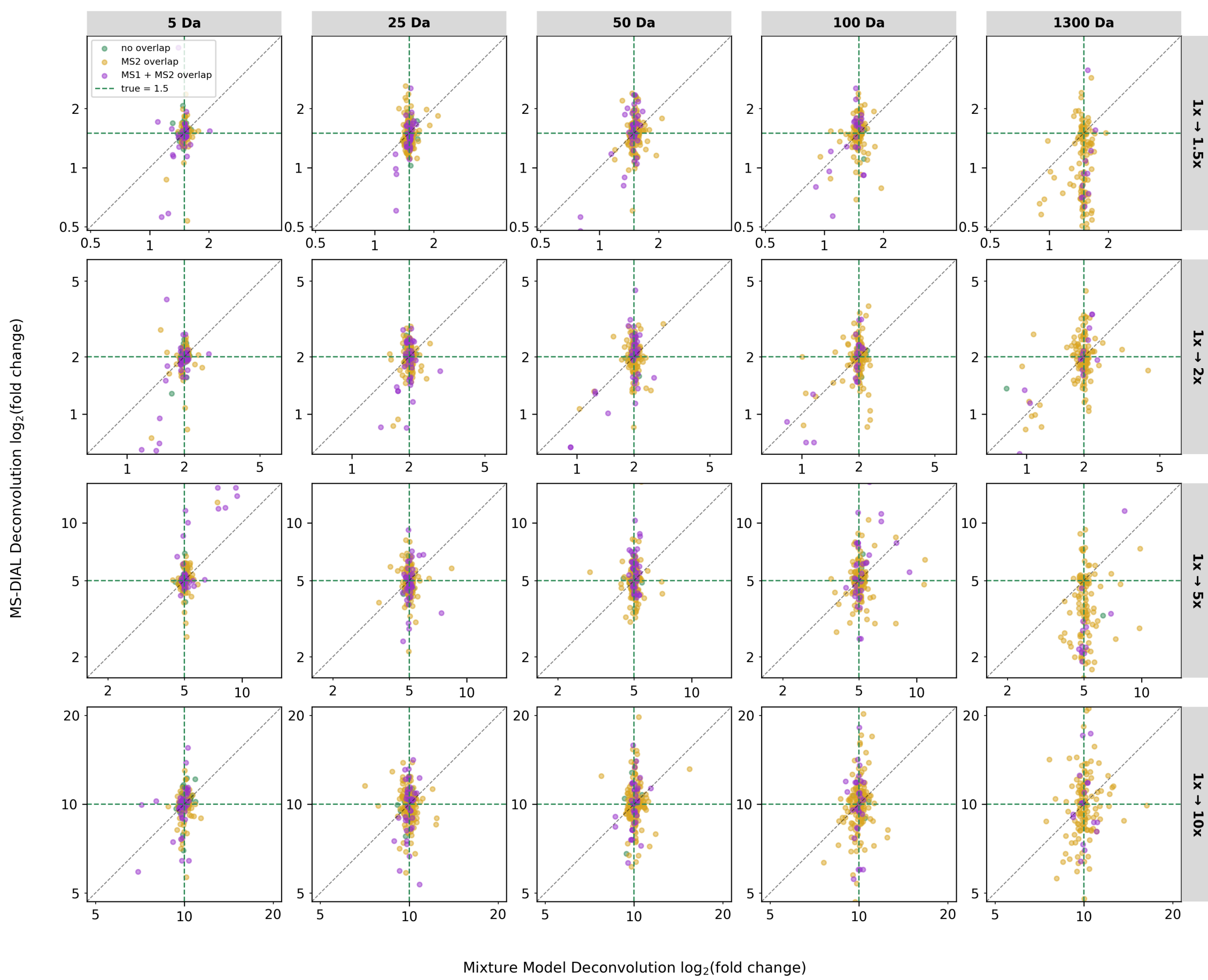

Figure S12: Precursor fold change reconstruction comparison between MMD and MS-DIAL. Points close to the vertical green dashed line mean that MMD has successfully recovered the abundance, and points close to the horizontal green dashed line mean that MS-DIAL has successfully recovered the abundance.
